# Optimizing irrigation and nitrogen rate can enhance the grain yield in both main and ratoon rice crop

**DOI:** 10.3389/fpls.2025.1646424

**Published:** 2025-09-01

**Authors:** Qiang Zhang, Yu Han, Mengying Qi, Haoran Jing, Xuanli Nie, Guilong Yu, Zhi Li, Pengfei Wei, Haiying Zhao, Mengjie Li, Hao Li, Tao Zhu

**Affiliations:** ^1^ College of Life Science and Engineering, Henan University of Urban Construction, Pingdingshan, Henan, China; ^2^ Xinyang Academy of Agricultural Sciences, Xinyang, Henan, China

**Keywords:** ratoon rice, irrigation regime, nitrogen application rates, yield, soil chemical properties

## Abstract

Ratoon rice enhances yield per unit area through efficient resource use, but the interactive effects of irrigation regime and nitrogen (N) rate on its productivity remain unclear. A two-year field experiment was conducted to assess the effects of two irrigation regimes—conventional flooding irrigation (CF) and alternate wetting and moderate soil drying irrigation (WMD)—combined with five N application rates on yield, agronomic productivity, root oxidation activity (ROA), soil chemical properties, and N use efficiency (NUE) in the rice cultivar Liangyou 6326. The results showed that compared with CF, WMD increased spikelets number per panicle, total spikelet number, and filled grain rate in both main and ratoon crop, thereby enhancing the yields by 11.0% and 16.1%, respectively. These improvements were linked to elevated soil N, phosphorus, potassium content, and cation exchange capacity under WMD, which enhanced photosynthesis, ROA and dry matter production. While the interaction between N rates and irrigation regimes on yield were nonsignificant, CF exhibited declining photosynthetic capacity, yield, and NUE with reduced N. Conversely, WMD showed initial increases followed by declines, peaking at 427.5 kg N/ha (243 kg ha^-1^ main crop; 184.5 kg ha^-1^ ratoon crop). This study provides actionable strategies for sustainable ratoon rice systems by balancing water and N inputs.

## 1 Introduction

Ratoon rice, a “one planting, two harvests” cultivation model, is an effective approach to increasing grain yield per unit area in southern China, particularly in regions with insufficient thermal resources for double-season rice production (Peng et al., 2023). Compared with single cropping rice, ratoon rice increased the multiple cropping index per unit area, raising yields by 75%–129% and increasing profits by more than 120%. In contrast to double cropping rice, it eliminated the need for seedling cultivation and transplanting, reducing costs by 32%–42% and boosting profits by more than 100% (Yuan et al., 2019). Thus, ratoon rice stands as a proven farming pattern for enhancing both rice production and economic returns in southern China. In recent years, the breeding of high regenerative capacity rice varieties and the development of supporting cultivation techniques have significantly improved ratoon rice yields and expanded its planting area (Shen et al., 2021). Currently, the planting area of ratoon rice in China exceeds 1 million ha, with a potential suitable area of over 13.28 million ha (Yu et al., 2022). Therefore, optimizing high yield and high efficiency cultivation techniques for ratoon rice is crucial for ensuring national food security.

Irrigation method and nitrogen (N) application rates are the main cultivation measures affecting rice growth and grain yield (Tao et al., 2024). Rational irrigation practices alter soil physicochemical properties and root physiological activity, thereby modulating the N absorption and utilization in crop production (Jin et al., 2024). As a novel water-saving irrigation technique, alternate wetting and moderate drying irrigation (WMD) not only increased rice yield and N utilization efficiency (NUE) but also enhanced water use efficiency (improving water use efficiency by over 20%), reduced irrigation water consumption in rice paddies (decreasing water usage by 25%–50%), and is now widely adopted in rice production (Djaman et al., 2018; Htwe et al., 2021; Gharsallah et al., 2023). Xu et al. (2017) reported that WMD enhances chlorophyll content, N concentration, net photosynthetic rate, and photosystem II efficiency in rice leaves, leading to higher yields when combined with moderate N application (240 kg ha^-1^). Wu et al. (2022) reported that compared with conventional flooding irrigation (CF), WMD increased the NUE by improving the N translocation and contribution rate in the stem sheath and leaf from heading to grain filling period of rice, allowing a 20% reduction in N input without compromising yield. Zheng et al. (2019) found that compared with CF, WMD during the whole growth period of main and ratoon crop not only reduced irrigation water amount but also reduced N application rate by 24% respectively, achieving water saving and N reduction goals. The enhanced NUE under WMD irrigation, which promotes rice growth and yield, can be attributed to the following mechanisms: (1) Improvement in soil physicochemical properties and fertility, optimizing nutrient availability and root zone conditions; (2) Modulation of soil microbial communities, including increased proportions of N fixing and nitrifying bacteria, coupled with elevated soil enzyme activity. These changes accelerate nutrient mineralization and mobilization, ensuring adequate nutrient supply for grain filling and ultimately boosting rice productivity (He et al., 2016). However, there are also studies showing that under CF, rice yield, aboveground N accumulation, N agronomic utilization rate, N absorption utilization rate, N partial productivity and N physiological utilization rate were significantly higher than those in WMD treatment, and rice yield was significantly improved when N was reduced by 25% under CF (Chen et al., 2012). These results demonstrate that the water-N interaction exhibits context dependent variability under distinct cultivation practices and agroecological conditions. Consequently, systematic exploration of irrigation regimes and N fertilization strategies is essential to elucidate their synergistic roles in regulating ratoon rice productivity and sustainability.

However, current research on the interactive effects of irrigation regimes and N fertilization in ratoon rice production remains limited, particularly regarding the impacts of irrigation N interactions throughout the entire growth cycle of both the main and ratoon crops on grain yield, NUE, and soil physicochemical properties. To address this knowledge gap, this study implemented two distinct irrigation regimes and five N application rates. The objectives were to elucidate the effects of irrigation and N management on the growth, development, and yield of ratoon rice, as well as to un-cover the underlying physiological mechanisms. The findings aim to provide a theoretical foundation and practical guidance for high-yield and resource efficient cultivation of ratoon rice.

## 2 Materials and methods

### 2.1 Experimental site and materials

The experiment was conducted at the Hudong Experimental Base of Xinyang Academy of Agricultural Sciences (in Xinyang, Henan Province, North China) in 2022 and 2023. The city is characterized by a temperate semi-humid monsoon climate, with an annual average temperature of 15.3°C (ranging from a maximum of 39°C to a minimum of -9°C). The frost-free period spans 220–230 days annually. The region receives an average annual precipitation of 1,100 mm, with an average relative humidity of 77% and annual sunshine duration ranging from 1,900 to 2,173 hours. The weather conditions in 2022 and 2023 are similar to the long-term average conditions (**Supplementary Figure S1**).

The soil was clay with a pH of 6.4, containing 54.3 mg kg^-1^ alkaline hydrolyzable nitrogen, 9.7 mg kg^-1^ available potassium, 79.8 mg kg^-1^ available phosphorus, and 11.4 g kg^-1^g organic matter. The tested rice variety was Liangyou 6326. Liangyou 6326 is an indica type two lines hybrid rice variety developed from the cross between Xuan 69S (a male sterile line) and Zhongxian WH26. This variety was released by the Xuancheng Agricultural Science Research Institute and passed the National Crop Variety Approval in 2007 [National Approved Rice 2007013]. Characterized by its strong regenerative ability, optimal growth duration, and superior grain quality, it currently serves as the primary ratoon rice cultivar widely cultivated across Henan and Hubei provinces.

### 2.2 Experimental design

**Figure 1:**
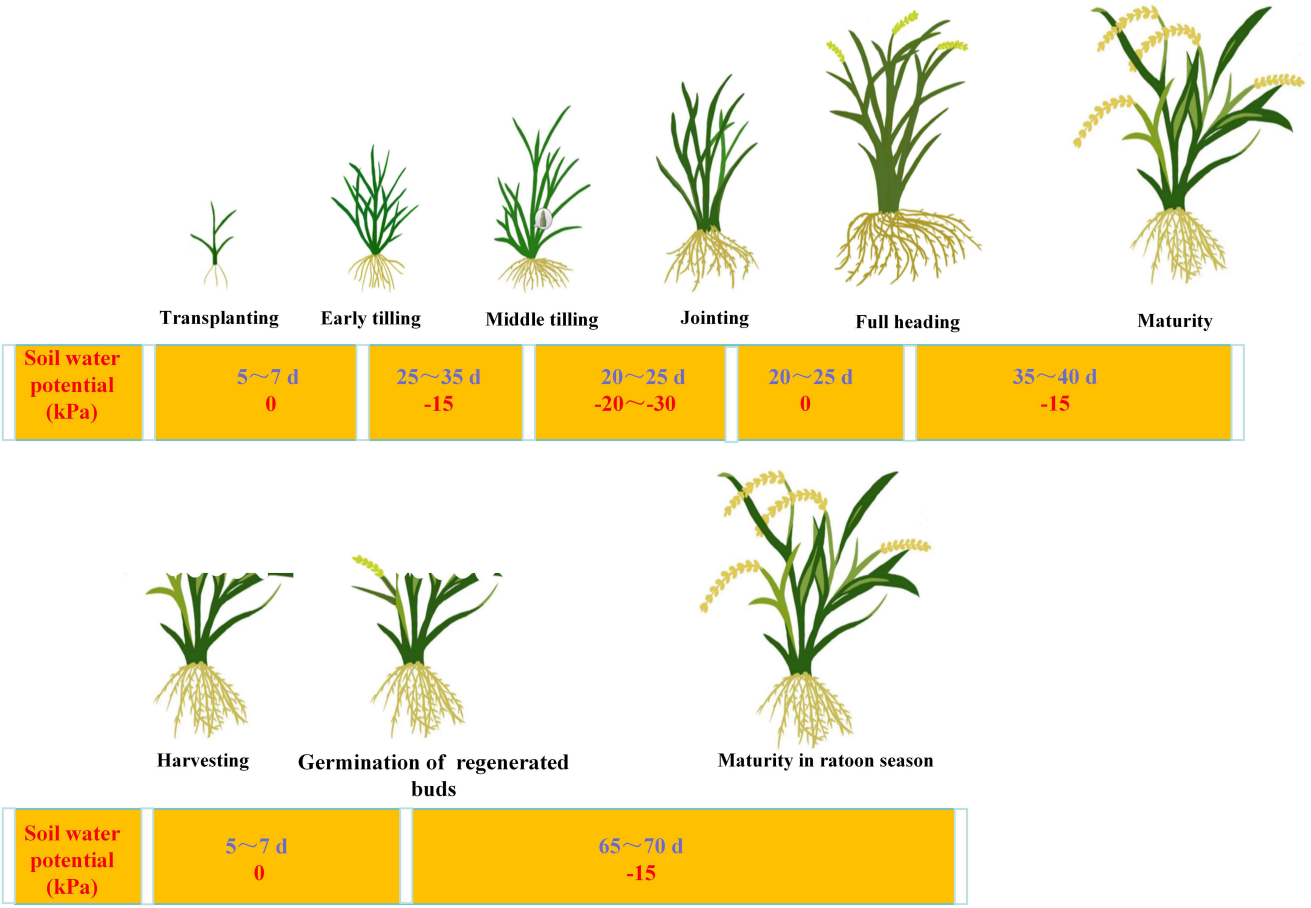
Irrigation regime of alternate wetting and moderate soil drying irrigation during the both two seasons of ratoon rice.

Sowing was conducted on March 4, 2022 and 2023, with the seedlings growing in a greenhouse and transplanting on April 15. The hill spacing was 0.33 by 0.15 m, with 2 seedlings per hill. The experiment was arranged in a split-plot design with three replications. Water regime served as the main-plot factor, and N application rate served as the sub-plot factor. The total number of experimental plots was 33, each measuring 4 m × 5 m. The irrigation treatments were designed as follows:

CF served as the control, maintaining a 2–3 cm water layer throughout the growth period of two seasons except for mid-season drainage during the ineffective tillering stage of the main crop. WMD was applied during the entire growth cycle of the two seasons (**Figure 1**). Soil water potential sensors (produced by Nanjing Institute of Soil Science, Chinese Academy of Sciences) were installed in the field at the 15–20 cm depth to monitor real time soil moisture. Each plot was equipped with three water potential sensors, and readings were recorded at 12.00 h each day. The zero-point calibration was performed by allowing the degassed sensors ceramic tip to dry naturally until the negative pressure reached 20 kPa, then vertically immersing it in water with the water level maintained at its midpoint, and recording the stabilized reading on the vacuum gauge as the calibration value. Irrigation (2–3 cm water layer) was triggered when soil water potential reached predefined thresholds, followed by natural drainage. This cycle was repeated to ensure optimal soil moisture conditions. N treatments comprised five application rates: N_100_ (270 N kg ha^-1^ in the main crop + 205 N kg ha^-1^ in the ratoon crop), N_90_ (243 N kg ha^-1^ in the main crop + 184.5 N kg ha^-1^ in the ratoon crop, N_80_ (216 N kg ha^-1^ in the main crop + 164 N kg ha^-1^ in the ratoon crop), N_70_ (189 N kg ha^-1^ in the main crop + 143.5 N kg ha^-1^ in the ratoon crop), N_60_ (162 N kg ha^-1^ in the main crop+123 N kg ha^-1^ in the ratoon crop). A zero N control (N_0_) was also included. Phosphorus (P) and potassium (K) fertilizers were uniformly applied as basal fertilizers across all plots. For N management, 60% of the total N in the main crop was allocated to basal and tillering fertilizers, with equal splits applied pre-transplanting and 5 days post-transplanting. The remaining 40% was applied as panicle fertilizers in two equal splits at the inverted 4th and 2nd leaf stages. In the ratoon crop, N fertilizers (bud promoting and seedling promoting) were applied at a 2:1 ratio—15 days after heading of the main crop and within 3 days postharvest of the ratoon rice. N, P, and K were applied to the field as urea (46% N), calcium superphosphate (12% P_2_;O_5_), and potassium chloride (60% K_2_;O), respectively. The specific fertilizer application rates are detailed in **Table 1**. Throughout the entire rice growth period, each experimental plot was covered with a steel-framed rain shelter clad in transparent plastic film (perimeter height: 3.5 m; top height: 5 m). The shelter was closed during rainfall and retracted during rain-free periods to minimize interference from natural precipitation. Field management, including pest, disease, and weed control, followed high yield cultivation protocols. In both 2022 and 2023, the main crops were harvested on August 15, and a post-harvest stubble height of 0.45 m was retained.

**Table 1:** N application rates in different treatments.

Treatment	Basal fertilizer	Tiller fertilizer	Panicle fertilizer	Panicle fertilizer	Budding fertilizer	Seedling fertilize
	Kg ha^-1^					
N_0_	0	0	0	0	0	0
N_100_	81.0	81.0	54.0	54.0	136.7	68.3
N_90_	72.9	72.9	48.6	48.6	123.0	61.5
N_80_	64.8	64.8	43.2	43.2	109.3	54.7
N_70_	56.7	56.7	37.8	37.8	95.7	47.8
N_60_	48.6	48.6	32.4	32.4	82.0	41.0

### 2.3 Sampling and measurements

#### 2.3.1 Recording the growth stage

The sowing date, transplanting date, jointing stage, full heading stage, and maturity stage were accurately recorded for both the main and ratoon crops.

#### 2.3.2 Dry matter weight and leaf area index

At the transplanting and jointing stages of the main crop, as well as the full heading and maturity stages of both the main and ratoon crops, representative plants form three hills were sampled from three points along the diagonal of each plot, based on the average growth status of the entire plot. Leaf area was measured using the length-width coefficient method. All sampled plants were separated into leaves, stems (including sheaths), and panicles (post-heading stage). The samples were oven-dried at 105°C for 30 minutes, then dried to constant weight at 70°C for DMW determination.

#### 2.3.3 Root oxidation activity

At the maturity stages of the main and ratoon crop, three representative hills per plot were sampled by excavating 20 cm × 20 cm × 20 cm soil blocks centered on rice bases. Roots were enclosed in 70-mesh sieve bags, rinsed with running water, and subsampled for determining ROA (Ramasamy et al., 1997). Briefly, Fresh root samples (1 g) were incubated with 50 mL of 20 mg L^-1^ α-NA in 150 mL conical flasks for 3 h with shaking. Subsequently, 2 mL aliquots of the extract were transferred to reaction vessels, mixed with 1 mL of 1% (w/v) sulfanilic acid and 1 mL of 0.1 g L^-1^ NaNO_3_, and thoroughly vortexed. α-NA content was determined spectrophotometrically (UV-1800, Shimadzu) after color development.

#### 2.3.4 Soil chemical properties

At the maturity stage of ratoon crop, soil samples (0–20 cm depth) were collected from each plot using an “S”-shaped sampling method (five replicates per plot). After collection, samples were air-dried naturally, sieved (2 mm mesh), and stored for analysis. Alkaline hydrolyzable N was determined by the alkali diffusion method; available phosphorus (P) by the Olsen method; available potassium (K) by NH_4_OAc extraction-flame photometry; cation exchange capacity (CEC) by the ammonium acetate exchange method; and soil pH by the potentiometric method with a soil-to-water ratio of 1:5 (w/v) (Zhu et al., 2024; Bai et al., 2020).

#### 2.3.5 Yield and its components

At maturity of both two seasons, plants from 1 m^2^ in each plot were sampled for panicle number and spikelet per panicle investigation. Filled grains were isolated using a flotation method where all spikelets were immersed in tap water after threshing to separate filled seeds from unfilled grains. A 1000-grain weight was measured by weighing three replicates of 1000 filled grains, with a permissible error ≤0.05 g. The grain yields of both two seasons were determined by harvesting and threshing separately from 5 m^2^ in each plot.

### 2.4 Data analysis

This study utilized Microsoft Office 2017 for text editing, data organization, and table formatting. SigmaPlot 11.0 and R 4.3.0 were employed for graphical visualization, while SPSS 25.0 was used for statistical analyses. The Least Significant Difference (LSD) method at P = 0.05 and P = 0.01 were applied to test the significance of mean differences. The following formulas were adopted:


(1) $$\text{Photosynthetic potential}(10^{4}{\text{ m}}^{2}\cdot \text{d}/\text{ha}):\text{PP}=\frac{\left({\text{L}}_{1}+{\text{L}}_{2})({\text{t}}_{2}-{\text{t}}_{1}\right)}{2}$$

(2) $$\text{Crop growth rate g}=({\text{m}}^{2}\cdot \text{d}):\text{CGR}=\frac{{\text{W}}_{2}-{\text{W}}_{1}}{{\text{t}}_{2}-{\text{t}}_{1}}$$

(3) $$\text{Net assimilation rate}[\text{g}=(\text{m}\cdot \text{d})]:\text{NAR}=\frac{{\text{lnLAI}}_{2}-{\text{lnLAI}}_{1}}{{\text{LAI}}_{2}-{\text{LAI}}_{1}}\times \frac{{\text{W}}_{2}-{\text{W}}_{1}}{{\text{t}}_{2}-{\text{t}}_{1}}$$

(4) $$\text{Dry matter accumulation}(\text{kg}):\text{DWA}={\text{W}}_{2}-{\text{W}}_{1}$$

(5) $$\text{Nitrogen partial factor productivity}(\text{kg}=\text{kg}):\text{NPFP}=\frac{{\text{Y}}_{\text{N}}}{{\text{N}}_{\text{rate}}}$$

(6) $$\text{Nitrogen agronomic efficiency}(\text{kg}/\text{kg}):\text{NAE}=\frac{{\text{Y}}_{\text{N}}-{\text{Y}}_{0}}{{\text{N}}_{\text{rate}}}$$

(Y_N_ and Y_0_: Grain yield of different N application rates and zero-N control; L_1_ and L_2_: Leaf area at the first and second measurements, respectively; t_1_ and t_2_: Time points of the first and second measurements; W_1_ and W_2_: Dry matter weight at the first and second measurements; LAI_1_and LAI_2_: Leaf area index at the first and second measurements, respectively).

## 3 Results

### 3.1 Interannual variance analysis

The yields of both the main and ratoon crops, as well as DMW, ROA and LAI at key growth stages, showed no significant differences between the two experimental years (2022 and 2023) but exhibited highly significant variations among water and N treatments. Similarly, the content of N, P, K and CEC in soil displayed no interannual significance but reached highly significant differences across treatments. Due to the consistent trends between the two years (**Supplementary Tables S1**–**S3**), data from the 2023 trial are presented in the main text, while results from the 2022 trial are provided in the Supplementary Materials.

### 3.2 Effects of irrigation regime and N application rate on dual-season grain yield

Both irrigation regime and N treatment significantly influenced the yield of the main crop. Under the same N level, compared to CF, WMD increased spikelets per panicle, total spikelet number, filled grain rate, and 1000-grain weight by averages of 2.0%, 3.4%, 4.7%, and 2.6%, respectively, resulting in an 11.0% overall yield enhancement (**Table 2**). Reducing N rates decreased panicle number, spikelets per panicle, and total spikelet number under CF and WMD irrigation regimes but increased filled grain rate and 1,000-grain weight. With N rates decreasing, the grain yield under CF declined steadily, while under WMD, it initially increased and then decreased, peaking at the N_90_ treatment (243 kg N/ha in the main crop).

**Table 2:** Effects of irrigation regime and N application rate on the grain yield of main crop.

Treatment		Panicles (10^4^ ha^-1^)	Spike panicle^-1^	Total spikelets (10^6^ha^-1^)	Filled grain rate (%)	1000-grain weight (g)	Grain yield (t ha^-1^)
WMD	N_100_	305.78±5.33a	160.33±2.38a	490.24±15.83a	71.61±1.09ef	28.50±0.42cd	10.01±0.32ab
	N_90_	302.40±5.50a	158.45±3.02ab	479.15±0.44a	75.81±0.60c	28.87±0.19cd	10.49±0.14a
	N_80_	284.33±1.32bc	152.92±0.82bc	434.78±0.31bc	77.40±1.14b	29.00±0.55bc	9.76±0.05b
	N_70_	253.50±9.70d	148.21±3.89d	375.72±24.23d	77.42±0.11b	29.81±0.29a	8.67±0.63de
	N_60_	239.03±3.88e	141.25±1.20e	337.63±2.61e	78.78±0.26a	29.89±0.26a	7.95±0.04fg
	**Mean**	**277.01±0.74A**	**152.23±0.98A**	**423.50±1.18A**	**76.21±0.36A**	**29.21±0.12A**	**9.38±0.03A**
CF	N_100_	306.00±4.70a	158.93±2.71a	486.32±15.76a	70.61±0.55f	27.96±0.38e	9.60±0.37bc
	N_90_	291.45±4.65b	154.69±3.14ab	450.84±1.96b	72.22±0.57de	28.30±0.24e	9.21±0.19cd
	N_80_	275.85±5.25c	150.69±1.57cd	415.68±3.59c	72.20±1.01de	28.20±0.21e	8.46±0.11ef
	N_70_	252.00±3.35d	147.84±4.28d	372.55±15.73d	73.31±0.54d	28.40±0.04de	7.76±0.28g
	N_60_	240.98±4.74e	134.04±4.88e	323.00±18.11e	75.57±0.72c	29.50±0.15ab	7.20±0.37h
	**Mean**	**273.26±0.78A**	**149.24±0.74B**	**409.68±3.78B**	**72.78±0.16B**	**28.47±0.06B**	**8.45±0.07B**
I		18.71*	399.41**	84.59*	133.18**	86.28*	1486.39**
N		142.17**	40.63**	128.65**	53.21**	17.86**	53.19**
I×N		1.42	1.01	0.84	6.46**	2.16	1.82

CF, conventional flooding; WMD, alternate wetting and moderate soil drying irrigation. N_100_, N_90_, N_80_, N_70_ and N_60_ represent 100%, 90%, 80%, 70% and 60% N application rates, respectively. I, irrigation regime; N, N application rate. Data in a column followed by different lower-case letters indicate significant differences at the 5% probability level according to the LSD test. Bold text indicates the mean values across different N application rate. Means followed by different upper-case letters indicate significant differences between the three nitrogen fertilizer levels at the 5% probability level according to the LSD test. ** represents the significant difference at the 1% level according to LSD test, * represents the significant difference at the 5% level according to LSD test.

In the ratoon crop, yield and its components followed similar trends (**Table 3**). Under the same N level, compared to CF, WMD increased panicle number, grains per spikelet, and total spikelet number by averages of 9.9%, 3.5% and 13.6%, respectively, resulting in an 16.1% overall yield enhancement. With decreasing N rates under the same irrigation regime, panicle number, spikelets per panicle, and total spikelets number gradually declined, while filled grain rate and 1,000-grain weight increased. Yield under CF exhibited a progressive reduction, whereas under WMD, it initially increased and then decreased, peaking at the N_90_ treatment (184.5 kg N/ha in the ratoon crop). ANOVA confirmed significant impacts (P < 0.05) of irrigation and N on panicle number, grains per spikelet, total spikelet number, and yield of ratoon-season. But their interaction was significant only for panicle number.

**Table 3:** Effects of irrigation regimes and N application rate on the grain yield of ratoon crop.

Treatment		Panicles (10^4^ ha^-1^)	Spike panicle^-1^	Total spikelets (10^6^ ha^-1^)	Filled grain rate (%)	1000-grain weight (g)	Grain yield (t ha^-1^)
WMD	N_100_	441.00±9.24a	68.09±0.45a	300.26±8.29a	65.67±0.87ef	27.62±1.08a	5.45±0.01a
	N_90_	434.40±2.10a	66.80±0.73a	290.16±1.75a	67.65±0.90de	27.80±0.78a	5.46±0.05a
	N_80_	406.05±4.54b	62.61±2.36b	254.22±6.73c	68.18±0.37de	28.01±0.39a	4.85±0.17b
	N_70_	357.23±5.17d	60.33±1.52bcd	215.52±2.30d	72.74±1.19c	28.23±0.39a	4.43±0.04bcd
	N_60_	311.70±8.55e	57.37±2.81d	178.82±3.87e	79.37±1.19a	28.62±0.20a	4.06±0.06de
	**Mean**	**390.08±3.01A**	**63.04±0.49A**	**247.80±0.35A**	**70.72±0.43A**	**28.06±0.41A**	**4.85±0.04A**
CF	N_100_	413.48±9.78b	66.83±1.71a	276.33±13.62b	63.69±2.82f	27.43±1.80a	4.83±0.77b
	N_90_	390.15±0.90c	63.64±0.88b	248.30±2.85c	66.34±1.61def	27.88±0.37a	4.59±0.10bc
	N_80_	353.18±2.32d	61.70±1.44bc	217.89±3.65d	68.80±1.55d	28.11±0.11a	4.21±0.15cde
	N_70_	318.23±5.97e	58.68±3.02cd	186.73±13.12e	71.55±3.00c	28.37±0.38a	3.79±0.16ef
	N_60_	300.83±6.73f	53.81±0.08e	161.86±3.85f	75.33±0.59b	28.38±0.57a	3.46±0.18f
	**Mean**	**355.17±2.39B**	**60.93±0.36B**	**218.22±0.71B**	**69.15±1.29A**	**28.03±0.34A**	**4.18±0.15B**
I		9816.49**	721.24**	20052.89**	9.94	0.01	47.01*
N		385.41**	35.46**	212.79**	67.21**	1.62	28.78**
I×N		9.96**	0.57	2.24	1.95	0.08	0.26

CF, conventional flooding; WMD, alternate wetting and moderate soil drying irrigation. N_100_, N_90_, N_80_, N_70_ and N_60_ represent 100%, 90%, 80%, 70% and 60% N application rates, respectively. I, irrigation regime; N, N application rate. Data in a column followed by different lower-case letters indicate significant differences at the 5% probability level according to the LSD test. Bold text indicates the mean values across different N application rate. Means followed by different upper-case letters indicate significant differences between the three nitrogen fertilizer levels at the 5% probability level according to the LSD test. ** represents the significant difference at the 1% level according to LSD test, * represents the significant difference at the 5% level according to LSD test.

### 3.3 Effects of irrigation regime and N application rate on photosynthetic capacity of ratoon rice

**Figure 2:**
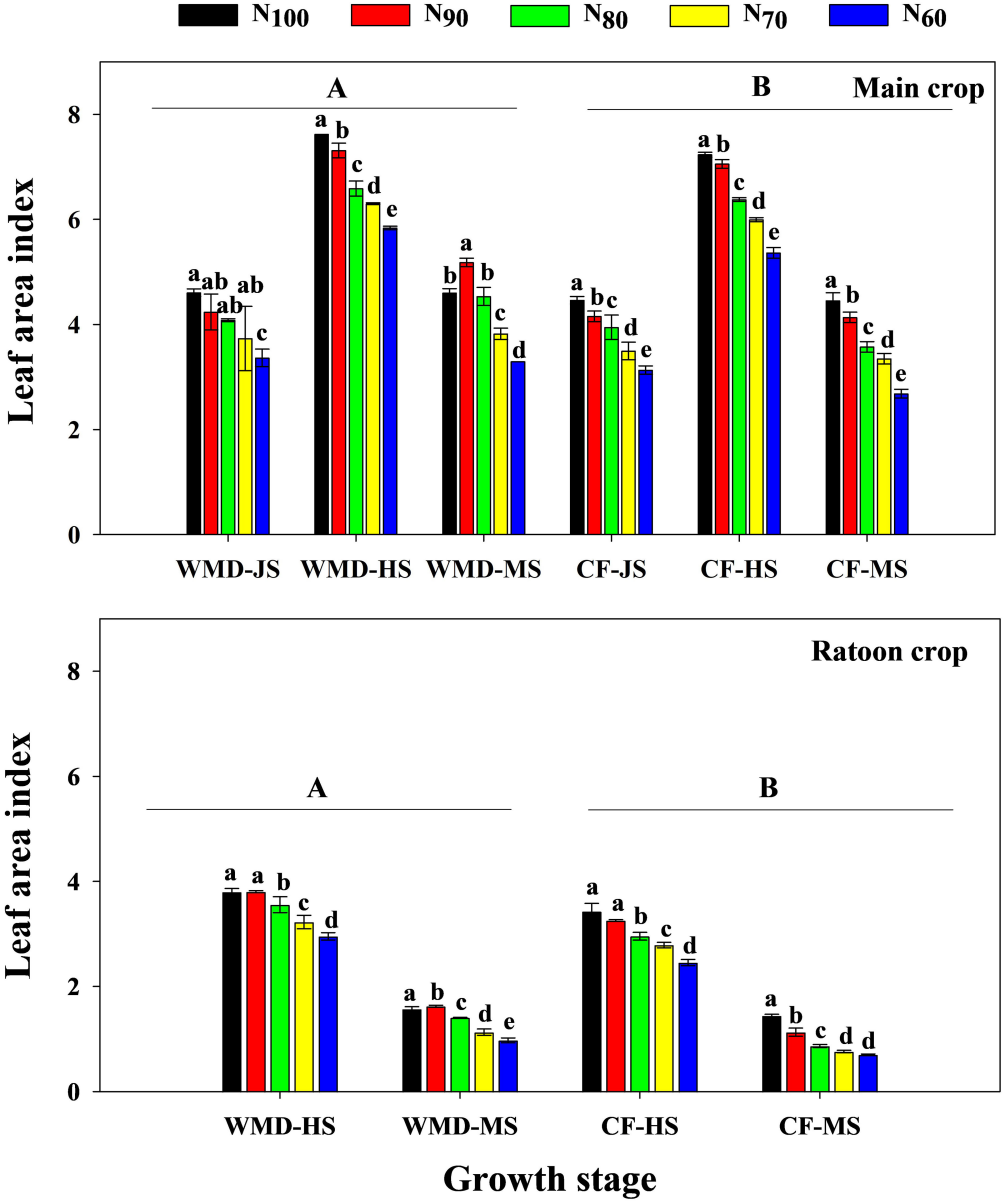
Effects of irrigation regime and N application rate on the LAI of main crop **(A)** and ratoon crop **(B)**. CF, conventional flooding; WMD, alternate wetting and moderate soil drying irrigation. N_100_, N_90_, N_80_, N_70_ and N_60_ represent 100%, 90%, 80%, 70% and 60% N application rates, respectively. JS, HS and MS represent jointing stage, full heading stage and maturity stage. Data in a same growth stage followed by different lower-case letters indicate significant differences at the 5% probability level according to the LSD test. Different lowercase letters indicate significant differences among treatments at same growth stage, and different capital letters indicate significant differences between irrigation regime (at the 5% probability level according to the LSD test).

**Figure 3:**
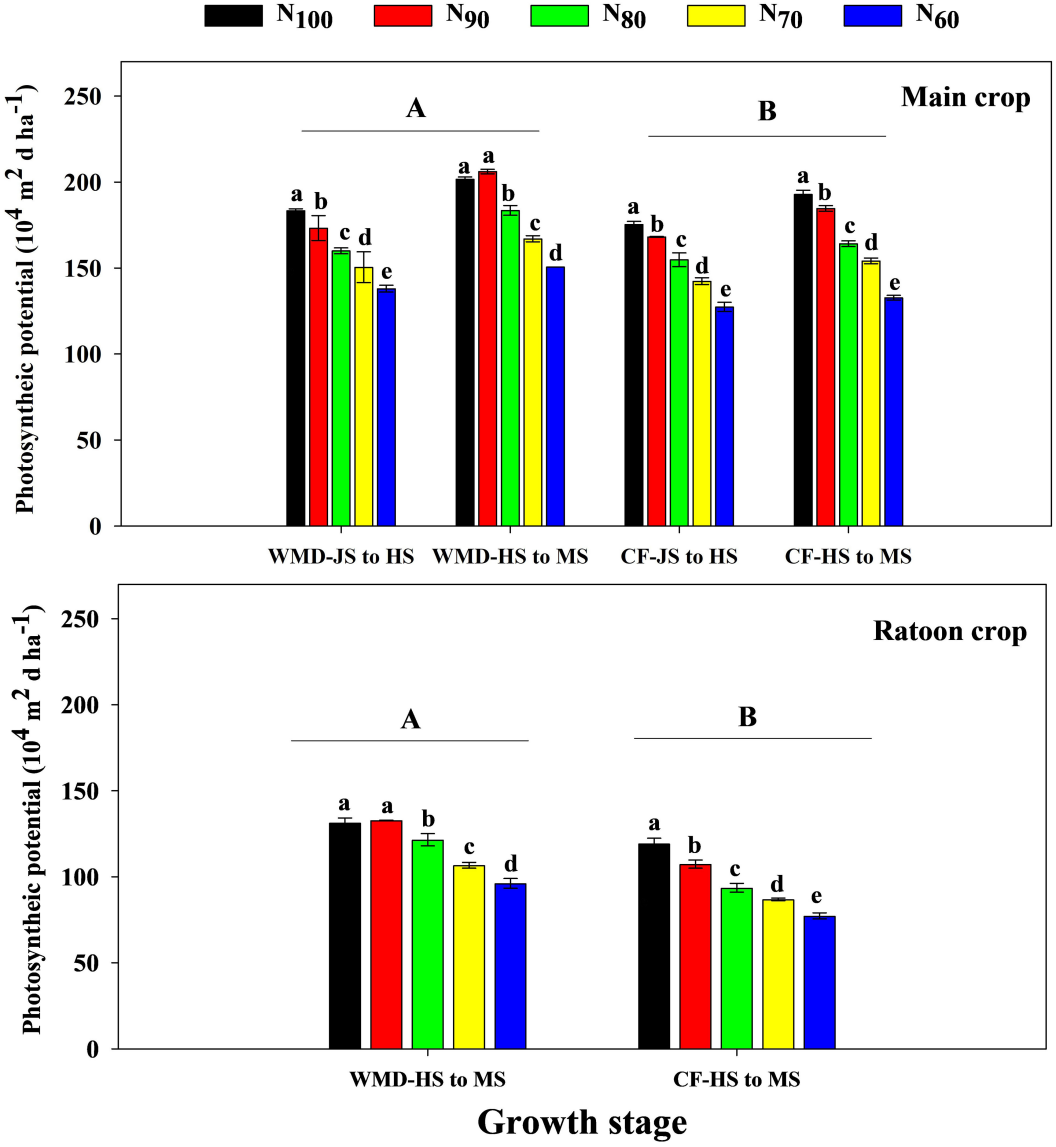
Effects of irrigation regime and N application rate on the PP of main crop **(A)** and ratoon crop **(B)**. CF, conventional flooding; WMD, alternate wetting and moderate soil drying irrigation. N_100_, N_90_, N_80_, N_70_ and N_60_ represent 100%, 90%, 80%, 70% and 60% N application rates, respectively. JS, HS and MS represent jointing stage, full heading stage and maturity stage. Different lowercase letters indicate significant differences among treatments at same growth stage, and different capital letters indicate significant differences between irrigation regime (at the 5% probability level according to the LSD test).

Under the same N level, compared to CF, WMD significantly increased the LAI of the main crop at jointing, full heading, and maturity stages by averages of 4.3%, 5.1%, and 17.8%, respectively (**Figure 2**). PP (Equation 1) of the main crop from jointing to heading and from full heading to maturity also increased significantly by 4.8% and 9.7%, respectively (**Figure 3**). With decreasing N rates, CF exhibited progressive reductions in LAI and PP of the main crop across all growth stages. In contrast, under WMD, LAI at jointing and full heading stages, as well as PP from jointing to full heading, gradually declined, while LAI at maturity and PP from heading to maturity showed an initial increase followed by a decline, peaking at the N_90_ treatment.

Similar trends were observed in the ratoon crop. Under same N levels, WMD significantly increased the LAI at heading and maturity stages by averages of 16.5% and 36.7%, compared to CF. PP from heading to maturity also significantly increased by 21.5%. Reducing N rates decreased LAI and PP across growth stages under CF, whereas under WMD, LAI at full heading stage declined gradually, but LAI at maturity and PP from heading to maturity exhibited an initial rise followed by a reduction, both reaching maxima at N_90_.

### 3.4 Effects of irrigation regime and N application rate on root oxidation activity of ratoon rice

**Figure 4:**
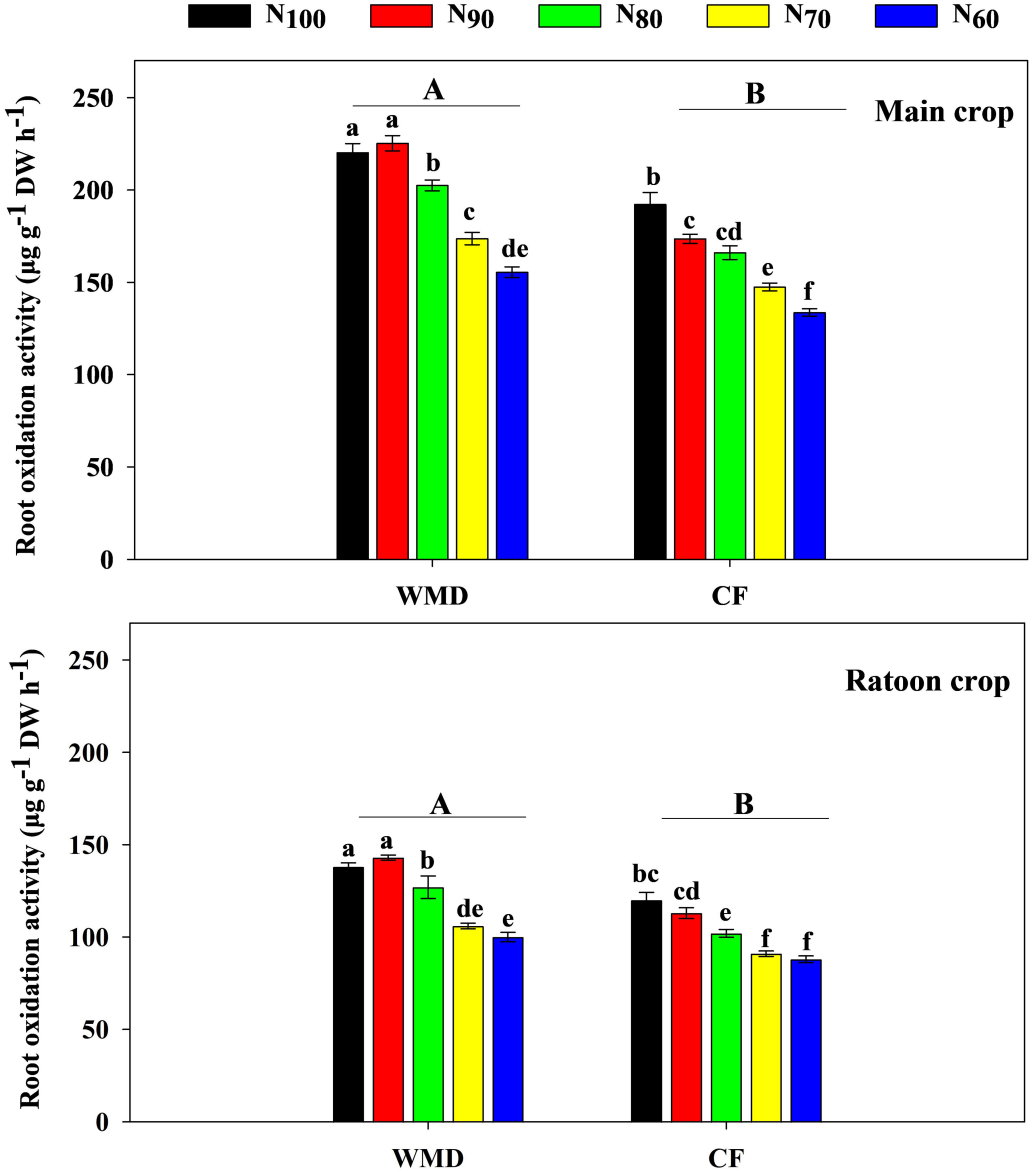
Effects of irrigation regime and N application rate on the ROA of main crop **(A)** and ratoon crop **(B)** in maturity stage. CF, conventional flooding; WMD, alternate wetting and moderate soil drying irrigation. N_100_, N_90_, N_80_, N_70_ and N_60_ represent 100%, 90%, 80%, 70% and 60% N application rates, respectively. Different lowercase letters indicate significant differences among treatments at same growth stage, and different capital letters indicate significant differences between irrigation regime (at the 5% probability level according to the LSD test).

Under same N levels, ROA at maturity in both main and ratoon crops was significantly higher in WMD than in CF, with average increases of 20.2% and 19.5%, respectively. As N application rate decreased, ROA gradually declined in CF during both seasons. In contrast, WMD exhibited an initial increase followed by a decrease, peaking at the N_90_ treatment (**Figure 4**).

### 3.5 Effects of irrigation regime and N application rate on CGR and NAR of ratoon rice

**Figure 5:**
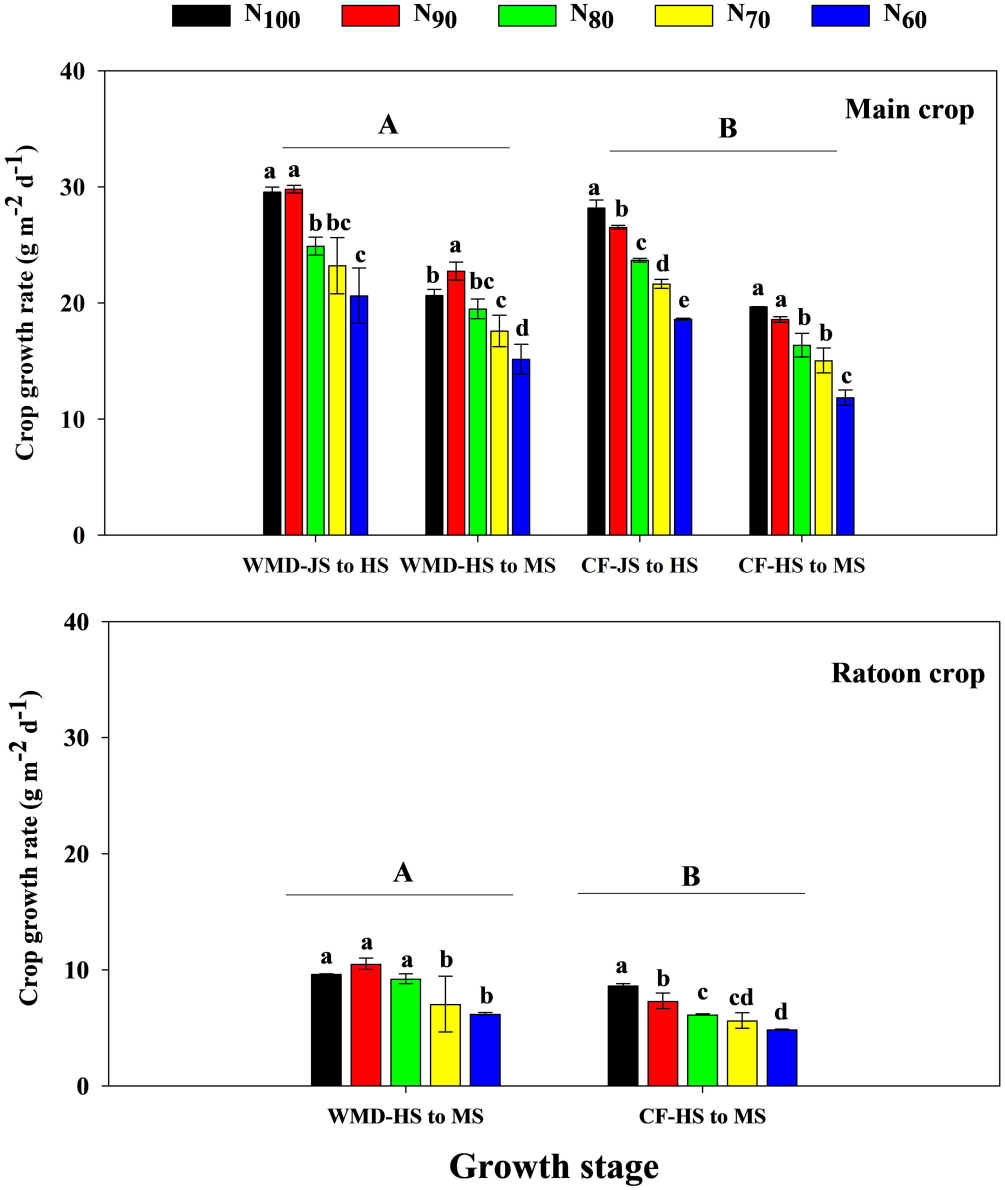
Effects of irrigation regime and N application rate on the CGR of main crop **(A)** and ratoon crop **(B)**. CF, conventional flooding; WMD, alternate wetting and moderate soil drying irrigation. N_100_, N_90_, N_80_, N_70_ and N_60_ represent 100%, 90%, 80%, 70% and 60% N application rates, respectively. JS, HS and MS represent jointing stage, full heading stage and maturity stage. Different lowercase letters indicate significant differences among treatments at same growth stage, and different capital letters indicate significant differences between irrigation regime (at the 5% probability level according to the LSD test).

**Figure 6:**
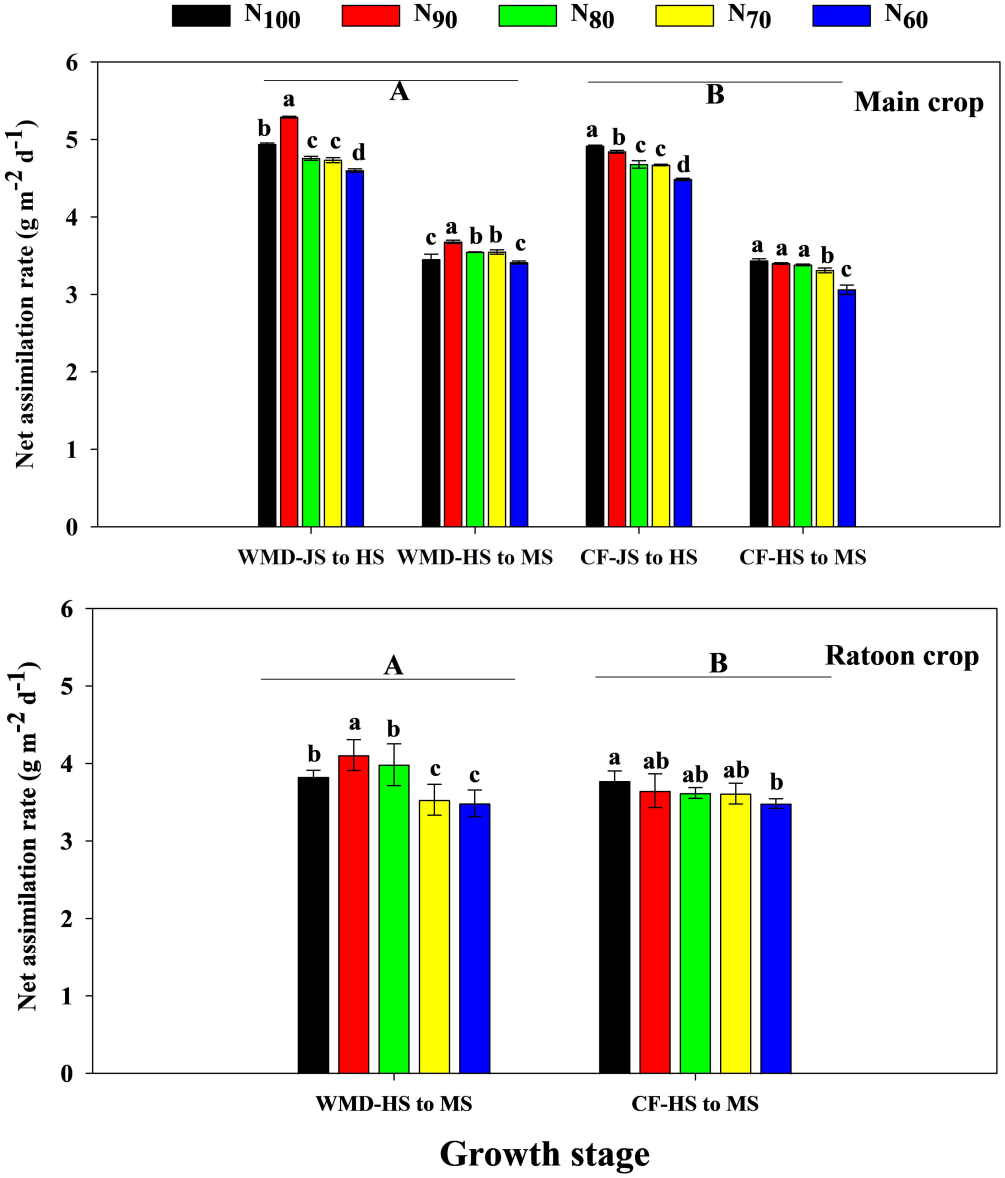
Effects of irrigation regime and N application rate on the NAR of main crop **(A)** and ratoon crop **(B)**. CF, conventional flooding; WMD, alternate wetting and moderate soil drying irrigation. N_100_, N_90_, N_80_, N_70_ and N_60_ represent 100%, 90%, 80%, 70% and 60% N application rates, respectively. JS, HS and MS represent jointing stage, full heading stage and maturity stage. Different lowercase letters indicate significant differences among treatments at same growth stage, and different capital letters indicate significant differences between irrigation regime (at the 5% probability level according to the LSD test).

Under the same N level, WMD significantly enhanced CGR (Equation 2) and NAR (Equation 3) of the main crop from jointing to full heading and full heading to maturity stages compared to CF, with average increases of 8.0% and 17.3% for CGR, and 3.1% and 6.3% for NAR, respectively (**Figures 5**, **6**). Reducing N rates led to gradual declines in CGR and NAR of the main crop across both growth phases under CF, whereas under WMD, these parameters initially increased and then decreased, reaching maxima at N_90_.

Similar trends were observed in the ratoon crop. Compared to CF, WMD increased CGR and NAR from full heading to maturity stages by 30.7% and 4.4%, respectively, under same N levels. With decreasing N rates, CF exhibited reductions in CGR and NAR from full heading to maturity stages. Conversely, under WMD, CGR and NAR from full heading to maturity stages displayed an initial increase followed by a decline, peaking at the N_90_ treatment.

### 3.6 Effects of irrigation regime and N application rate on DMW and DMA in ratoon rice

**Figure 7:**
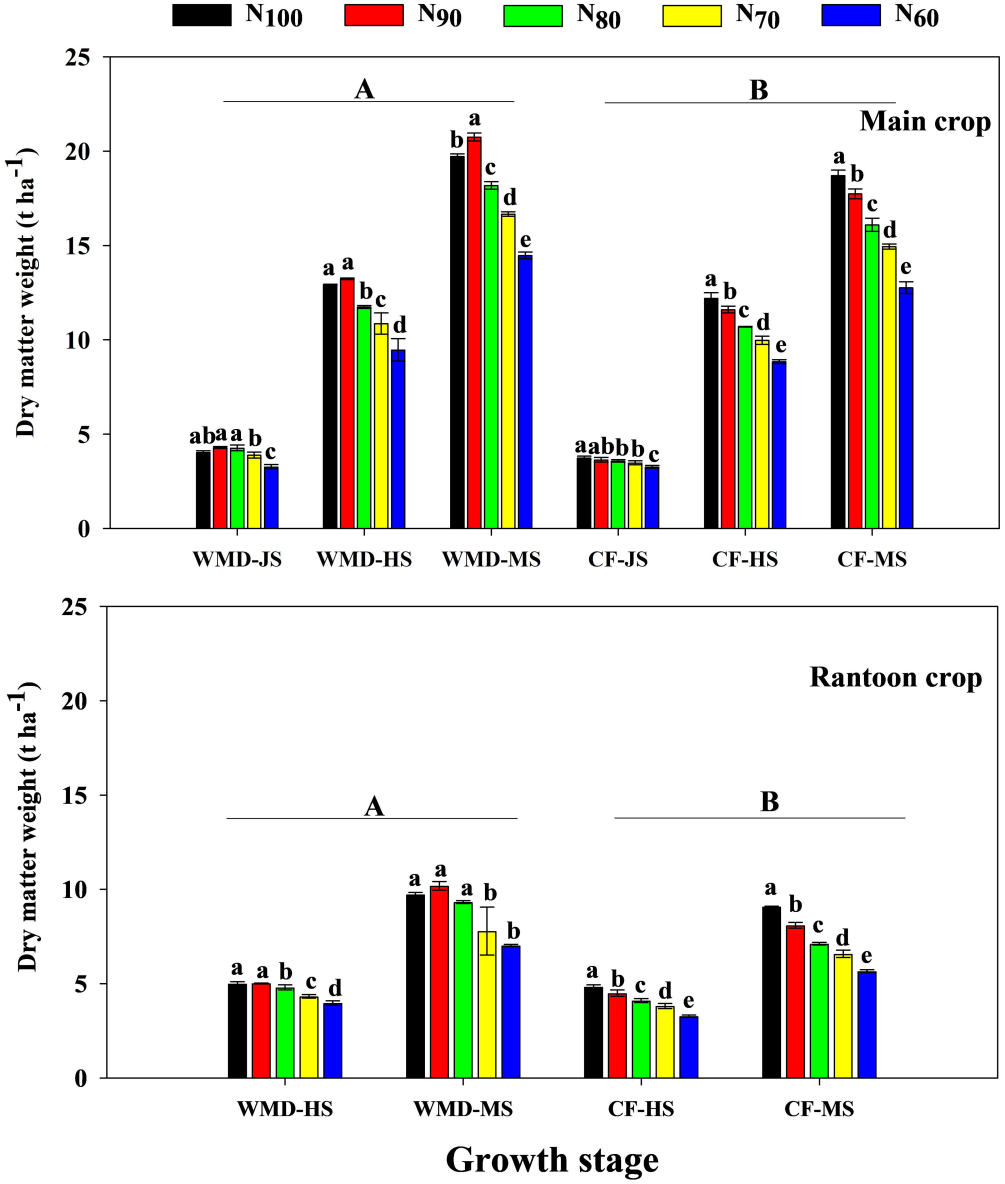
Effects of irrigation regime and N application rate on the DMW of main crop **(A)** and ratoon crop **(B)**. CF, conventional flooding; WMD, alternate wetting and moderate soil drying irrigation. N_100_, N_90_, N_80_, N_70_ and N_60_ represent 100%, 90%, 80%, 70% and 60% N application rates, respectively. JS, HS and MS represent jointing stage, full heading stage and maturity stage. Different lowercase letters indicate significant differences among treatments at same growth stage, and different capital letters indicate significant differences between irrigation regime (at the 5% probability level according to the LSD test).

**Figure 8:**
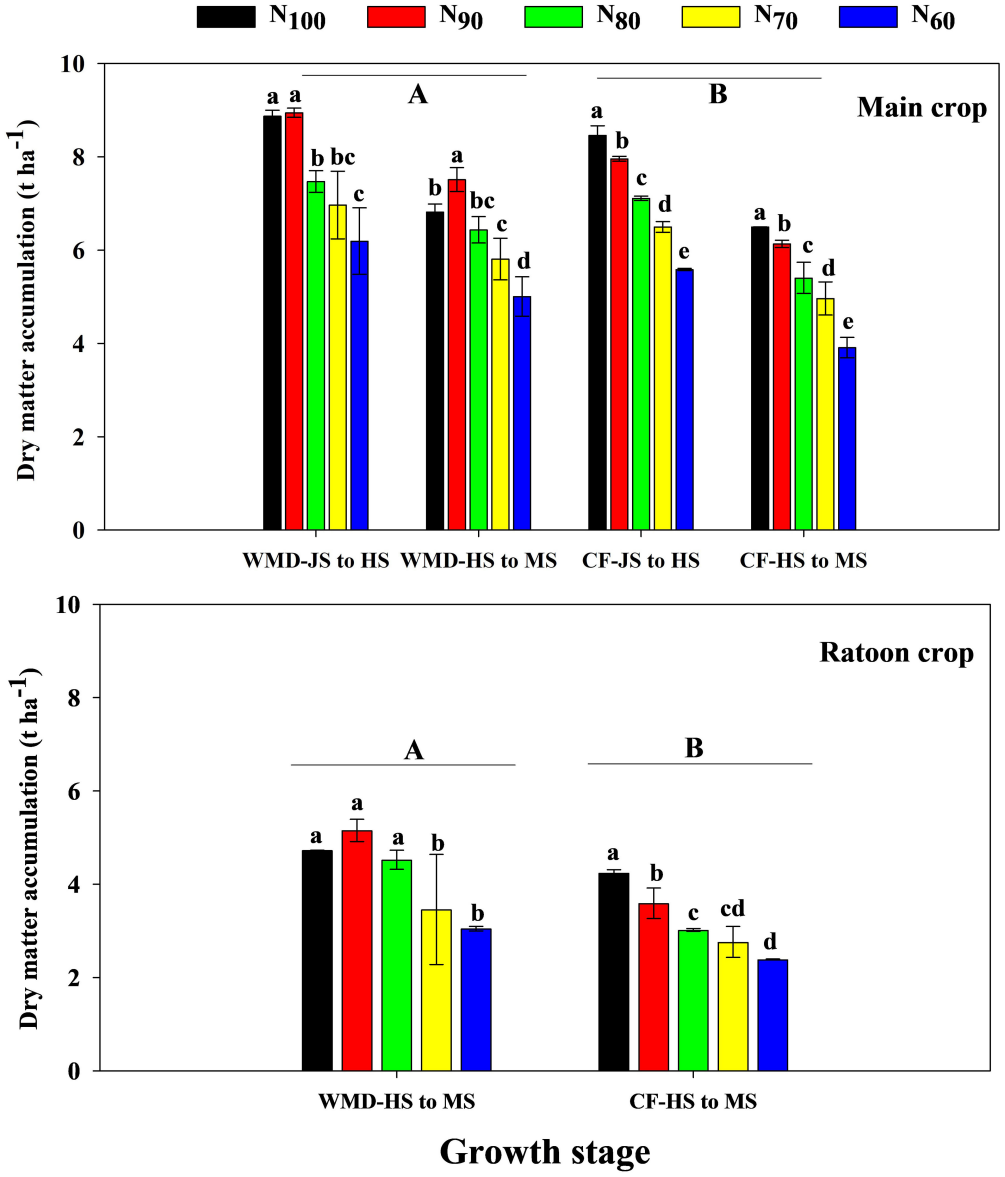
Effects of irrigation regime and N application rate on the DWA of main crop **(A)** and ratoon crop **(B)**. CF, conventional flooding; WMD, alternate wetting and moderate soil drying irrigation. N_100_, N_90_, N_80_, N_70_ and N_60_ represent 100%, 90%, 80%, 70% and 60% N application rates, respectively. JS, HS and MS represent jointing stage, full heading stage and maturity stage. Different lowercase letters indicate significant differences among treatments at same growth stage, and different capital letters indicate significant differences between irrigation regime (at the 5% probability level according to the LSD test).

Under the same N level, WMD significantly increased DMW of the main crop at jointing, full heading, and maturity stages by averages of 11.5%, 9.1%, and 11.9%, respectively, compared to CF (**Figure 7**). DMA (Equation 4) of the main crop from jointing to full heading and full heading to maturity was also significantly enhanced by 8.0% and 17.3%, respectively (**Figure 8**). Reducing N rates led to gradual decreases in DMW at all stages and DMA across growth phases under CF, whereas WMD showed a rise-then-decline pattern, with maxima consistently achieved at N_90_.

Similar trends were observed in the ratoon crop. WMD increased DMW at full heading, maturity, and DMA from heading to maturity by 12.7%, 20.6%, and 30.7%, respectively, compared to CF under same N levels. With reduced N rates, CF exhibited progressive declines in DMW at all stages and DMA from full heading to maturity. In contrast, WMD displayed an initial increase followed by a decline in these parameters, peaking at the N_90_ treatment. Reducing N rates led to gradual decreases in DMA under CF, whereas WMD showed a rise-then-decline pattern, with maxima consistently achieved at N_90_.

### 3.7 Effects of irrigation regime and N application rate on soil chemical properties

Irrigation regimes and N application rate significantly influenced paddy soil chemical properties (**Table 4**). Under the same N level, WMD significantly increased soil alkaline hydrolyzable N, available P, available K, CEC, and pH by averages of 20.9%, 19.3%, 5.8%, 12.3%, and 5.7%, respectively, compared to CF. With decreasing N rates, alkaline hydrolyzable N, available P, available K, and CEC progressively declined under both CF and WMD, while pH remained stable. ANOVA indicated that irrigation regimes significantly affected all measured soil properties (P<0.05), whereas N rates exerted highly significant effects (P<0.01) on all parameters except pH. The interaction between irrigation regimes and N rates significantly influenced alkaline hydrolyzable N, available P, and available K (P<0.05).

**Table 4:** Effects of irrigation regime and N application rate on the soil chemical properties.

Treatment		N (mg/kg)	P (mg/kg)	K (mg/kg)	CEC (mol/kg)	pH
WMD	N_100_	100.76±0.94a	25.05±1.11a	160.22±1.00a	16.39±0.01a	7.18±0.01a
	N_90_	98.78±1.04a	24.52±0.27a	158.48±2.13a	16.00±0.27ab	7.21±0.01a
	N_80_	95.15±1.40b	23.01±0.93b	148.60±2.87b	15.72±0.42b	7.22±0.05a
	N_70_	92.13±1.08b	21.12±0.19c	148.60±3.75b	15.67±0.56b	7.15±0.07a
	N_60_	82.36±3.42d	19.78±0.31d	147.17±3.19b	15.10±0.52c	7.15±0.08a
	**Mean**	**93.84±0.60A**	**22.70±0.49A**	**152.61±2.59A**	**15.78±0.25A**	**7.18±0.02A**
CF	N_100_	85.85±0.70c	23.25±0.16b	157.22±1.87a	14.73±0.01cd	6.85±0.05b
	N_90_	78.17±4.19e	19.58±1.36d	142.85±0.62c	14.17±0.05d	6.77±0.09b
	N_80_	77.47±0.70e	18.47±0.16d	141.42±1.19c	14.07±0.02d	6.76±0.10b
	N_70_	76.78±1.95e	16.99±0.95e	139.98±2.75c	14.10±0.22d	6.79±0.02b
	N_60_	69.80±0.98f	16.81±0.13e	139.98±1.00c	13.18±0.01e	6.80±0.01b
	**Mean**	**77.61±0.64B**	**19.02±0.49B**	**144.29±0.74B**	**14.05±0.05B**	**6.79±0.01B**
I		4607.45**	43.92*	18.67*	273.93**	473.89**
N		66.24**	97.32**	125.26**	21.46**	0.71
I×N		3.81*	7.19**	16.28**	0.44	0.83

CF, conventional flooding; WMD, alternate wetting and moderate soil drying irrigation. N, P, K and CEC represent hydrolyzable N, available phosphorus, available potassium and cation exchange capacity, respectively. N_100_, N_90_, N_80_, N_70_ and N_60_ represent 100%, 90%, 80%, 70% and 60% N application rates, respectively. I, irrigation regime; N, N application rate. Data in a column followed by different lower-case letters indicate significant differences at the 5% probability level according to the LSD test. Bold text indicates the mean values across different N application rate. Means followed by different upper-case letters indicate significant differences between the three nitrogen fertilizer levels at the 5% probability level according to the LSD test. ** represents the significant difference at the 1% level according to LSD test, * represents the significant difference at the 5% level according to LSD test.

### 3.8 Relationship between ratoon rice yield and soil factors

**Figure 9:**
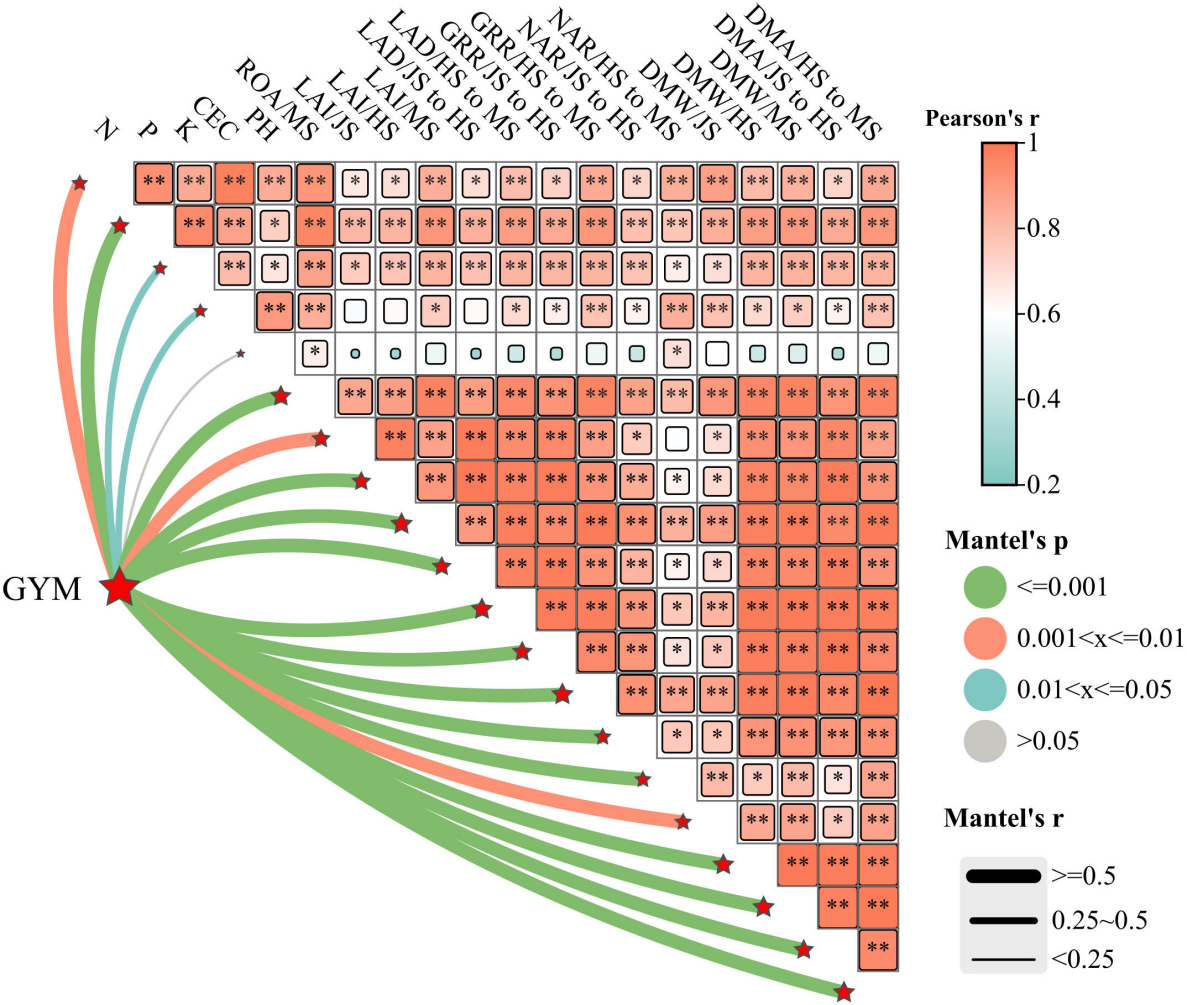
Correlation between grain yield of main crop and soil quality and growth index of main crop. GYM, grain yield of main crop; JS, HS and MS represent jointing stage, full heading stage and maturity stage, respectively. LAI, PP, CGR, NAR, DMW and DWA represent leaf area index, photosynthetic potential, crop growth rate, net assimilation rate, dry matter weight and dry matter accumulation, respectively. N, P, K and CEC represent the content of hydrolyzable nitrogen, available phosphorus, available potassium and cation exchange capacity, respectively. * and ** represent the significant difference at the 5% and 1% level according to LSD test, respectively.

**Figure 10:**
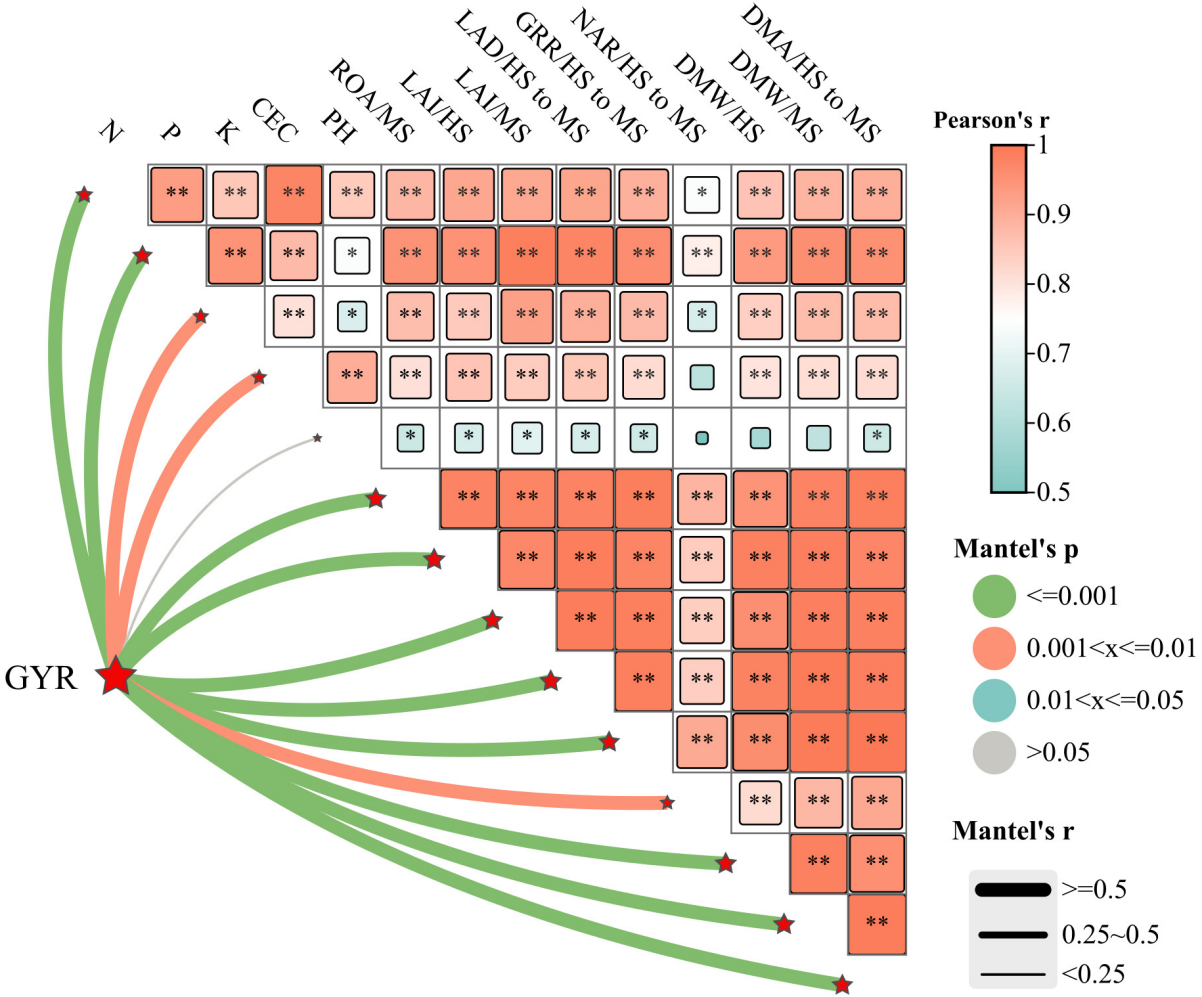
Correlation between grain yield of ratoon crop and soil quality and growth index of ratoon crop. GYR, grain yield of ratoon crop; JSH, S and MS represent jointing stage, full heading stage and maturity stage, respectively. LAI, PP, CGR, NAR, DMW and DWA represent leaf area index, photosynthetic potential, crop growth rate, net assimilation rate, dry matter weight and dry matter accumulation, respectively. N, P, K and CEC represent the content of hydrolyzable nitrogen, available phosphorus, available potassium and cation exchange capacity, respectively. * and ** represent the significant difference at the 5% and 1% level according to LSD test, respectively.

**Figure 11:**
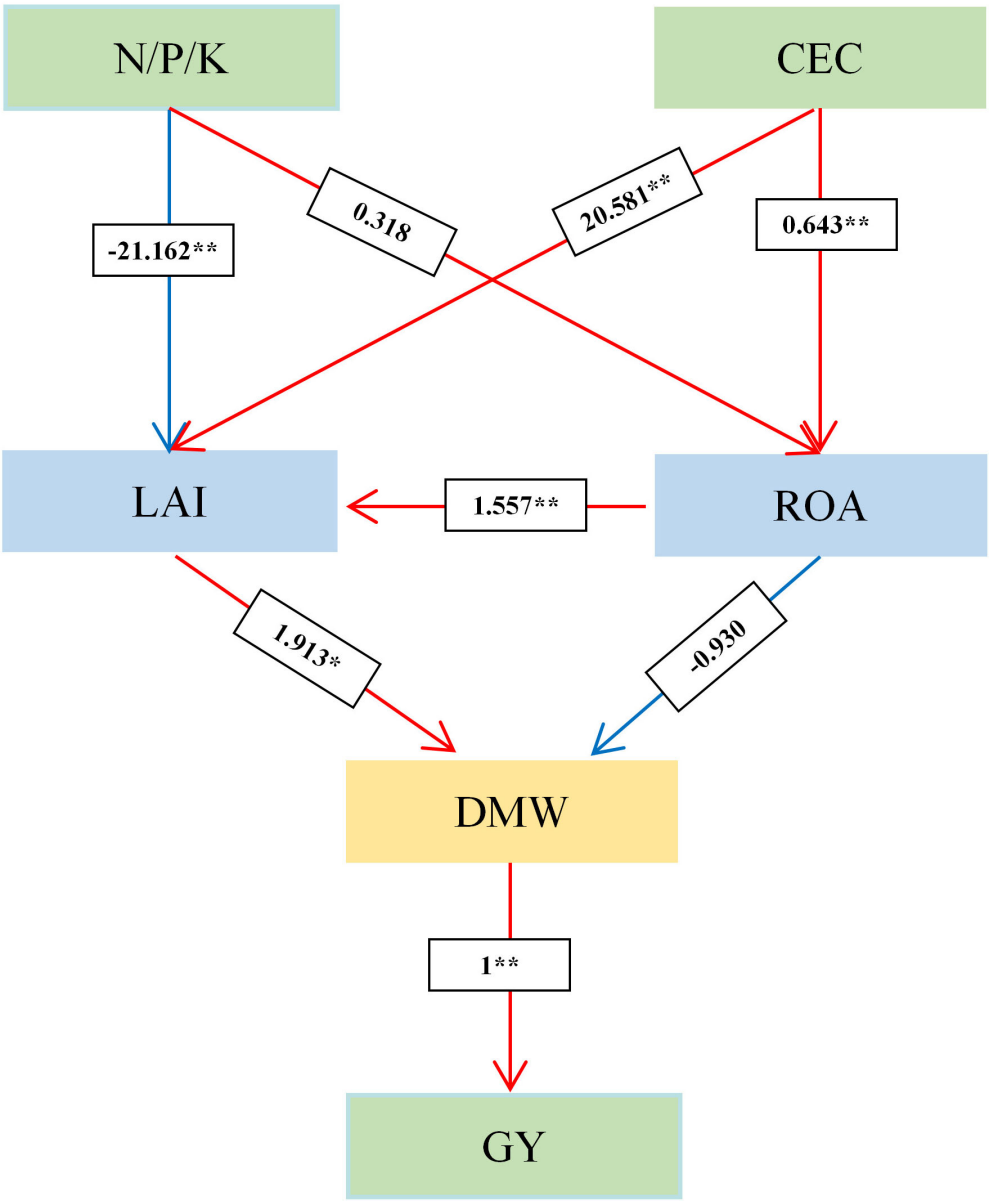
Structural equation modeling diagram of the effects of soil physicochemical properties on yield. *and ** indicate significance levels at *P*<0.05 and *P*<0.01, respectively; blue and red connections represent negative and positive relationships, respectively; values on the connections denote standardized path coefficients. N, P, K and CEC represent the content of hydrolyzable nitrogen, available phosphorus, available potassium and cation exchange capacity, respectively. LAI, ROA and DMW represent leaf area index, root oxidation activity, and dry matter weight, respectively.

Mantel analysis revealed that the main crop yield exhibited significant or highly significant positive correlations with soil nutrient content (N, P, K), CEC, LAI, PP, DMW, DMA, CGR, and NAR during the main crop (**Figure 9**). Similarly, ratoon crop yield showed analogous correlations with soil nutrients (N, P, K), CEC, LAI, PP, DMW, DMA, CGR, and NAR in the ratoon crop (**Figure 10**). Structural equation modeling analysis indicated that CEC and soil nutrient content (N, P, K) content significantly increased grain yield by enhancing DMW through improved LAI and ROA (**Figure 11**). These findings indicate that rational irrigation and fertilization management synergistically improved rice photosynthetic efficiency and dry matter production by enhancing soil nutrient availability and CEC, thereby significantly increasing yields of both two seasons in ratoon rice.

### 3.9 Effects of irrigation regime and N application rate on total N use efficiency of ratoon rice

Under the same N level, compared to CF, WMD significantly increased the NPFP (Equation 5) and NAE (Equation 6) by averages of 12.9% and 13.3%, respectively (Table 5). As N application rates decreased, NPFP increased, while NAE showed no consistent trend, with the highest NAE observed in the WMD-N_90_ treatment. ANOVA indicated that irrigation regimes had significantly affected NPFP (P<0.01) but not NAE. N application rates significantly affected NPFP (P<0.01) but not NAE. No significant interaction effects between irrigation regimes and N rates were detected for either NPFP or NAE.

## 4 Discussion

### 4.1 Effects of irrigation regime and N application rate on grain yield of both two seasons

Water and N are critical agronomic factors influencing rice growth and yield. Rational irrigation and N management can optimize crop population, enhance physiological processes, and significantly improve productivity. Extensive studies have demonstrated that WMD irrigation, as a water-saving practice, confers substantial yield advantages. The underlying mechanisms include: (1) WMD promoted the secretion of zeatin + zeatin riboside and indole-3-acetic acid, positively regulating shoot growth and development (Ha et al., 2012; Yang et al., 2000); (2) WMD enhanced chlorophyll content in leaves during key growth stages, thereby improving photosynthetic capacity and dry matter production (Fu et al., 2024); (3) WMD increased the activity of starch hydrolase enzymes in sheaths, facilitating nutrient translocation from stems to grains (Geng et al., 2023), while also upregulating key enzymes in sucrose-starch metabolism in inferior spikelets, promoting grain filling and increasing grain weight (Xu et al., 2018). Recently, WMD has been adapted to ratoon rice systems. Zhang et al. (2019) demonstrated that WMD applied throughout the main crop significantly increased yields in both seasons. Zhang et al. (2022) further reported that WMD increased soluble sugars and cytokinins in stems during the main crop, improving the growth and development of regenerated buds and increasing grain yield in ratoon crop. However, the physiological mechanisms by which applying WMD throughout dual-season improved grain yields of both two seasons remain unclear. Our results revealed that, compared to CF, WMD significantly increased spikelets per panicle, 1,000-grain weight, and filled grain rate in both seasons, leading to an 11.0% (main crop) and 16.1% (ratoon crop) yield improvement (**Tables 2**, **3**). The yield enhancing mechanism is attributed to increased ROA, LAI, PP, CGR and NAR during critical growth phases, which collectively enhance biomass production (**Figure 2**–**8**). Notably, WMD significantly enhanced ratoon crop yield more than main crop yield (**Table 2**, **3**). Similarly, increases in leaf photosynthetic capacity, crop growth rate, and dry matter accumulation were substantially greater in the ratoon crop (**Figures 2**, **5**, **8**). This differential effect likely arises because maintaining high root activity and photosynthetic capacity in the main crop after heading supported regenerated tiller growth and development since regenerated tillers sprout from main crop stems (Zhang et al., 2021; Peng et al., 2023). Thus, WMD in main crop not only improved main crop growth but also directly promoting regenerated tiller development and growth (Zhang et al., 2022). Furthermore, following regenerated tiller emergence, WMD in ratoon crop further stimulated regenerated seedling growth, ultimately leading to the larger increases in grain yield observed in the ratoon crop. These findings extend previous research and provide novel insights into high-yield ratoon rice cultivation.

N application rate effects on grain yield varied under different irrigation regimes. Under CF, reducing N rates progressively decreased photosynthetic capacity, dry matter production, and yield in both seasons. Conversely, under WMD, these parameters initially increased and then declined, peaking at N_90_ (**Figure 2**-**8**). This highlights a significant irrigation-N interaction. Optimal irrigation can reduce N demand in rice by improving nutrient availability (Zhang et al., 2013), while N modulates soil moisture dynamics and plant water uptake (Ishfaq et al., 2020). Xu et al. (2015) found that WMD enhanced root activity and N absorption, enabling high yields even at moderate N levels. However, excessive N under water deficit increased water stress and impaired the rice growth and development (Chu et al., 2017). Chu et al. (2017) reported significant interaction effects between irrigation regimes and N rates on rice yield and NUE, with WMD combined with moderate N application (160 kg ha^-1^) achieving the highest yield and NUE. Similarly, Fu et al. (2023) demonstrated that WMD coupled with 270 kg N/ha maximized rice yield, but exceeding this threshold led to yield reductions. Yang et al. (2021) further highlighted that irrigation-N interactions significantly influenced root growth, biomass allocation to roots, stems, leaves, and panicles, as well as canopy growth rate. Alternate partial root-zone drying irrigation combined with 165 kg N/ha was found to optimize both NUE and yield. Contrasting these findings, our ANOVA results indicated that irrigation-N interactions significantly affected only filled grain rate in main crop and panicle number in ratoon crop (P<0.05), with no significant impacts on total yield or other yield components (**Tables 2**, **3**). These discrepancies suggest that optimal irrigation-N synergies depend on cultivar-specific traits, environmental conditions, and cropping systems. Therefore, site-specific adjustments in irrigation and N management are critical to achieving water-N synergy and maximizing yield efficiency.

### 4.2 Effects of irrigation regime and N application rate on soil chemical properties in ratoon rice

Paddy soil, as the foundation for rice growth, critically influences crop development, yield formation, and grain quality. Previous studies have demonstrated that WMD irrigation significantly altered soil physical, chemical, and biological properties. Compared to CF, WMD enhanced soil porosity, reduced bulk density, improved soil aeration, and increased soil organic matter, available N, P, K, and CEC, thereby increasing rice yields (Peng et al., 2018). Consistent with these findings, our study revealed that WMD elevated soil alkaline hydrolyzable N, available P, available K, CEC, and pH compared to CF (**Table 4**). This effect may occur because, compared to CF, WMD enhances soil aeration. Consequently, this stimulated root growth, increased root exudate content and soil microbial activity, and elevated the activity of key soil enzymes (urease, catalase, invertase, acid phosphatase) (Jiang et al., 2021; He et al., 2023). These changes collectively accelerated soil nutrient mineralization and facilitated the release of available nutrients (alkaline hydrolyzable N, available P, available K). Furthermore, the enhanced microbial activity promoted organic matter accumulation and transformation, improving soil CEC and pH (Majumdar et al., 2023; Islam et al., 2025).

The enhanced soil nutrient availability under WMD provided sufficient resources for ratoon rice growth, ultimately improving grain yields in both two seasons. Correlation analysis further confirmed significant or highly significant positive correlations (P<0.05 or P<0.01) between key soil properties (alkaline hydrolyzable N, available P, available K, and CEC) and yield components in both two seasons (**Figures 9**, **10**). These soil parameters also exhibited strong associations with photosynthetic and dry matter production indices in the main and ratoon crops (**Figures 9**, **10**). These results collectively indicate that WMD improves soil chemical properties, which synergistically enhances photosynthetic efficiency and biomass accumulation, driving yield increases in both two seasons.

N application rate is another critical factor influencing soil physicochemical properties. Ge et al. (2013) reported that increasing N rates elevated soil organic matter, available P, and available K in tobacco fields but reduced available N. Similarly, Wang et al. (2012) found that N fertilization significantly increased soil organic matter and alkaline hydrolyzable N in paddy soils, though excessive N application (300 kg ha^-1^) had no significant improvement. Li (2023) demonstrated that higher N rates markedly increased nitrate and ammonium N contents in 0–120 cm soil layers under wheat-maize rotation systems. Our results also showed that increasing N rates progressively enhanced soil alkaline hydrolyzable N, available P, available K, and CEC in ratoon rice fields, while pH remained unaffected (**Table 4**). Studies have indicated potential interaction effects between irrigation regimes and N application rates on soil physicochemical properties. Chen et al. (2017) demonstrated that different water-fertilizer regimes in ratoon rice significantly influenced rhizosphere redox potential and soil enzyme activity. Specifically, WMD coupled with seedling promoting fertilizers during the tillering stage of ratoon crop markedly enhanced rhizosphere redox potential and enzyme activity. Wang et al. (2023) further reported that water-fertilizer coupling (360 mm irrigation + 120 kg N kg^-1^) significantly improved soil nutrient availability and enzymatic activity in Panax notoginseng fields. However, our findings revealed no statistically significant irrigation-N interaction effects on soil chemical properties (**Table 4**). This discrepancy may arise from variations in cropping systems, cultivar-specific responses, or environmental heterogeneity. Additionally, our focus on soil chemical properties excluding physical and biological traits may have limited the detection of interaction effects. Future studies should integrate assessments of soil structure, microbial communities, and enzymatic dynamics to comprehensively unravel water-nitrogen management impacts on soil health.

### 4.3 Effects of irrigation regime and N application rate on NUE in ratoon rice

Both irrigation regimes and N application rates significantly influence NUE in rice. Liu et al. (2009) demonstrated that WMD during the grain filling stage increased both AEN and physiological efficiency of N under high N application (300 kg ha^-1^) compared to CF. However, Atwill et al. (2018) reported no significant difference in NUE between WMD and CF. Our results revealed that WMD significantly improved NPFP and AEN in ratoon rice compared to CF (**Table 5**). The underlying mechanisms may include: (1) enhanced rhizosphere oxygen availability under WMD, which promotes root growth and activates key enzymes in N metabolism, thereby increasing N uptake (Xu et al., 2013; Ye et al., 2013); and (2) improved soil physicochemical properties, elevated soil N, P, K content, and enhanced urease activity, ensuring sufficient nutrient supply for ratoon rice growth (Yang et al., 2004).

**Table 5:** Effects of irrigation regime and N application rate on the total nitrogen utilization rate in two seasons.

Treatment		NPFP (g/g)	NAE (g/g)
WMD	N_100_	32.54±0.69fg	14.33±0.62bc
	N_90_	37.30±0.22e	17.06±0.22a
	N_80_	38.46±0.32e	15.70±0.32ab
	N_70_	49.24±2.23b	16.72±2.23a
	N_60_	52.69±0.40a	14.75±0.40bc
	**Mean**	**42.04±0.41A**	**15.71±0.41B**
CF	N_100_	30.38±0.85g	14.20±1.40bc
	N_90_	32.30±0.21fg	14.33±1.33bc
	N_80_	33.36±0.11f	13.15±1.46de
	N_70_	43.42±1.46d	14.54±2.69bc
	N_60_	46.79±2.42c	13.10±3.45e
	**Mean**	**37.25±0.26B**	**13.87±1.71B**
I		143.56**	13.24
N		215.78**	2.68
I×N		2.12	1.42

CF, conventional flooding; WMD, alternate wetting and moderate soil drying irrigation. NPFP, N partial factor productivity; NAE, N agronomic efficiency. N_100_, N_90_, N_80_, N_70_ and N_60_ represent 100%, 90%, 80%, 70% and 60% N application rates, respectively. I, irrigation regime; N, N application rate. Data in a column followed by different lower-case letters indicate significant differences at the 5% probability level according to the LSD test. Bold text indicates the mean values across different N application rate. Means followed by different upper-case letters indicate significant differences between the three nitrogen fertilizer levels at the 5% probability level according to the LSD test. ** represents the significant difference at the 1% level according to LSD test, * represents the significant difference at the 5% level according to LSD test.

Existing studies on irrigation-N interactions remain inconsistent. Chu et al. (2017) and Li and Yang (2017) reported significant interaction effects on NUE, whereas Pan et al. (2017) and Kar et al. (2015) observed no such interactions. Our findings indicated no significant irrigation-N interaction effects on NUE in ratoon rice production. However, the highest AEN was achieved under WMD combined with N_90_. These results suggest that integrating WMD with optimized N application (e.g., N_90_) not only enhances yield but also reduces N input and improves NUE, achieving dual objectives of productivity and sustainability in ratoon rice systems.

## 5 Conclusions

This study investigated the effects of irrigation regimes and N application rates on grain yield, soil chemical properties, and NUE in ratoon rice. The results demonstrated that, compared to CF, WMD irrigation applied throughout the entire growth of two seasons significantly improved dual-season yields and NUE. The yield-increasing mechanism were twofold: (1) increased soil nutrient content, and (2) promoted rice growth, which enhanced ROA, photosynthetic capacity and biomass production, thereby exhibiting significant advantages in yield improvement and resource efficiency. Furthermore, under WMD irrigation, excessive N fertilization proved detrimental to ratoon rice yield and NUE. The optimal total N application rate was identified as 427.5 kg ha^-1^ (243 kg ha^-1^ in the main crop + 184.5 kg ha^-1^ in the ratoon crop). This study confirms that optimized irrigation management combined with appropriate N fertilization effectively promotes the growth and development of both main and ratoon rice crops, thereby enhancing overall ratoon rice productivity.

## Data Availability

The original contributions presented in the study are included in the article/supplementary material. Further inquiries can be directed to the corresponding author.
