# Supplementary material for: Optimizing irrigation and nitrogen rate can enhance the grain yield in both main and ratoon rice crop
Source: Front Plant Sci. 2025 Sep 1;16:1646424. doi: 10.3389/fpls.2025.1646424 (PMC12434168; doi:10.3389/fpls.2025.1646424)
Supplement: Supplementary file 1 [file DataSheet1.docx]

Supplementary Material

# Supplementary Figures and Tables

| 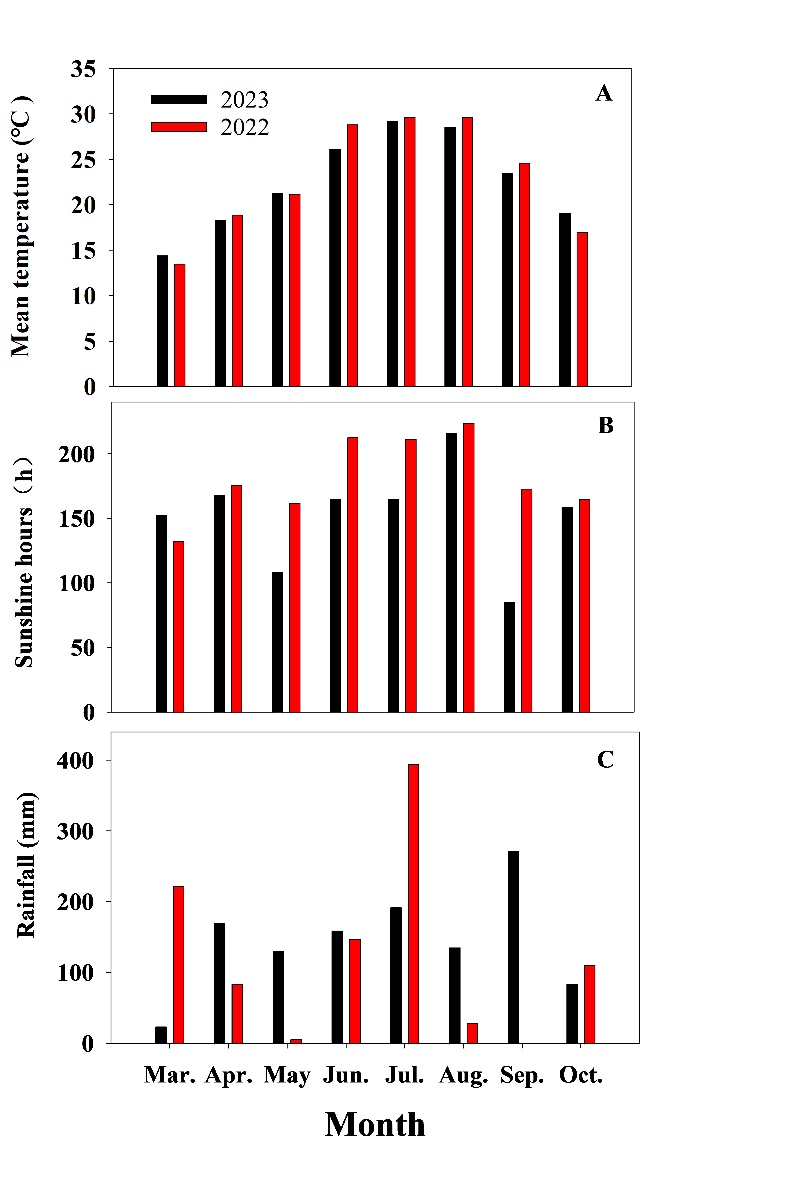 |
| --- |
| **Figure S1.** Mean temperature (A), sunshine hours (B) and rainfall (C) monthly during rice growth in 2022 and 2023. |

## Supplementary Figures

| 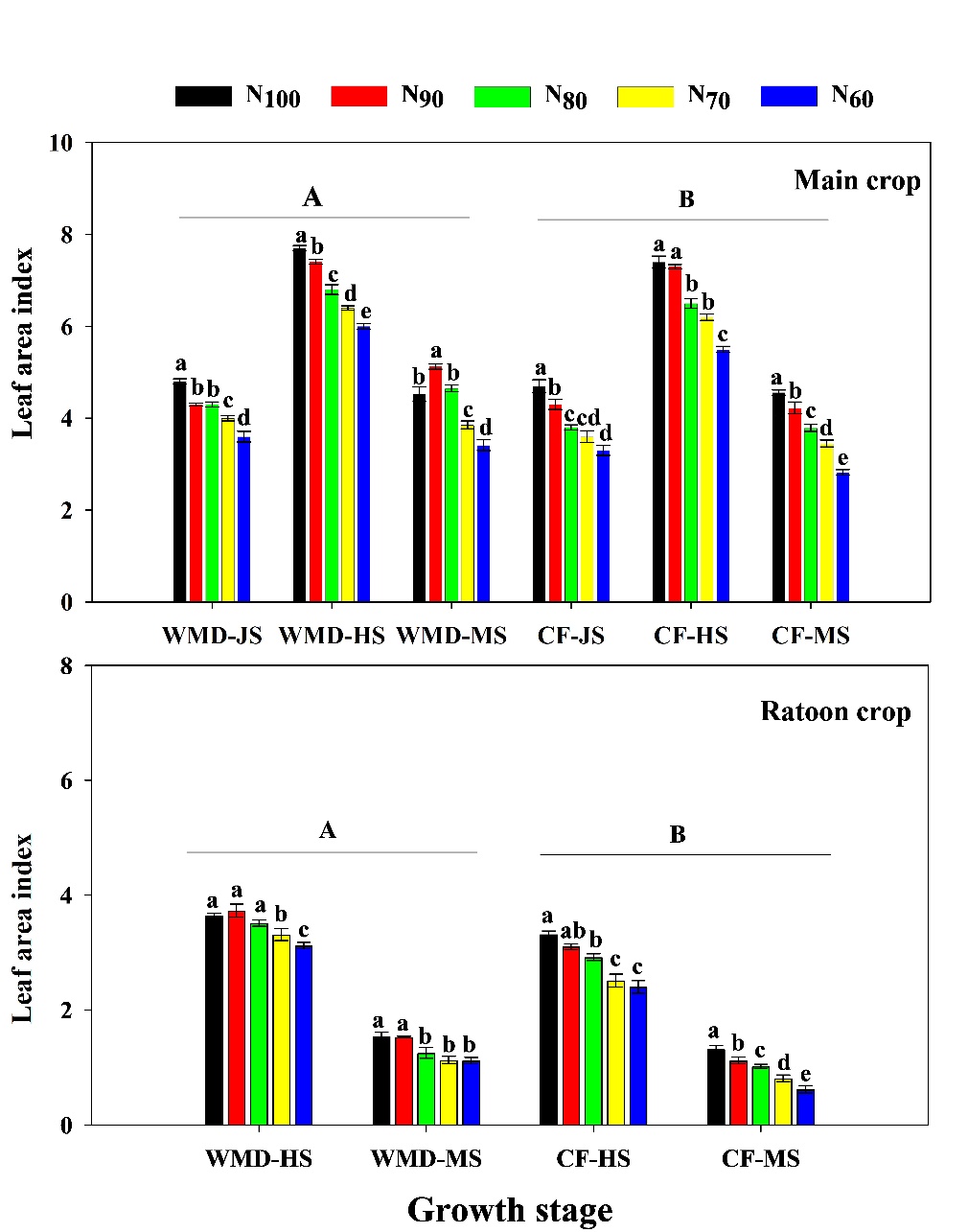 |
| --- |
| **FigureS2****.** Effects of irrigation regime and N application rate on the LAI of main crop (A) and ratoon crop (B) in 2022. Note: CF, conventional flooding; WMD, alternate wetting and moderate soil drying irrigation. N_100_, N_90_, N_80_, N_70_ and N_60_ represent 100%, 90%, 80%, 70% and 60% N application rates, respectively. JS, HS and MS represent jointing stage, full heading stage and maturity stage. Different lowercase letters indicate significant differences among treatments at same growth stage, and different capital letters indicate significant differences between irrigation regime (at the 5% probability level according to the LSD test). |

| 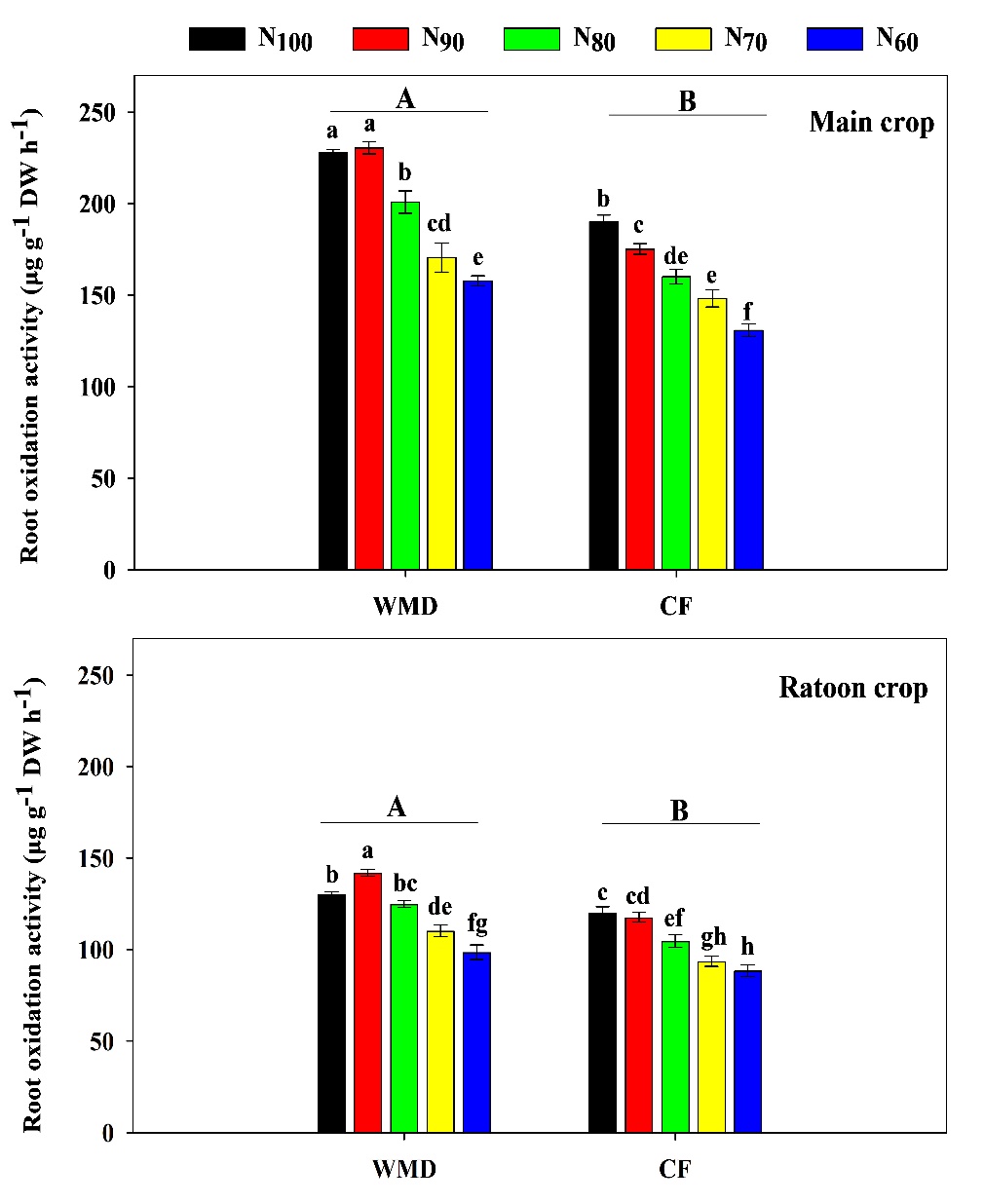 |
| --- |
| **FigureS3.** Effects of irrigation regime and N application rate on the ROA of main crop (A) and ratoon crop at maturity stage (B) in 2022. Note: CF, conventional flooding; WMD, alternate wetting and moderate soil drying irrigation. N_100_, N_90_, N_80_, N_70_ and N_60_ represent 100%, 90%, 80%, 70% and 60% N application rates, respectively. Different lowercase letters indicate significant differences among treatments at same growth stage, and different capital letters indicate significant differences between irrigation regime (at the 5% probability level according to the LSD test). |

| 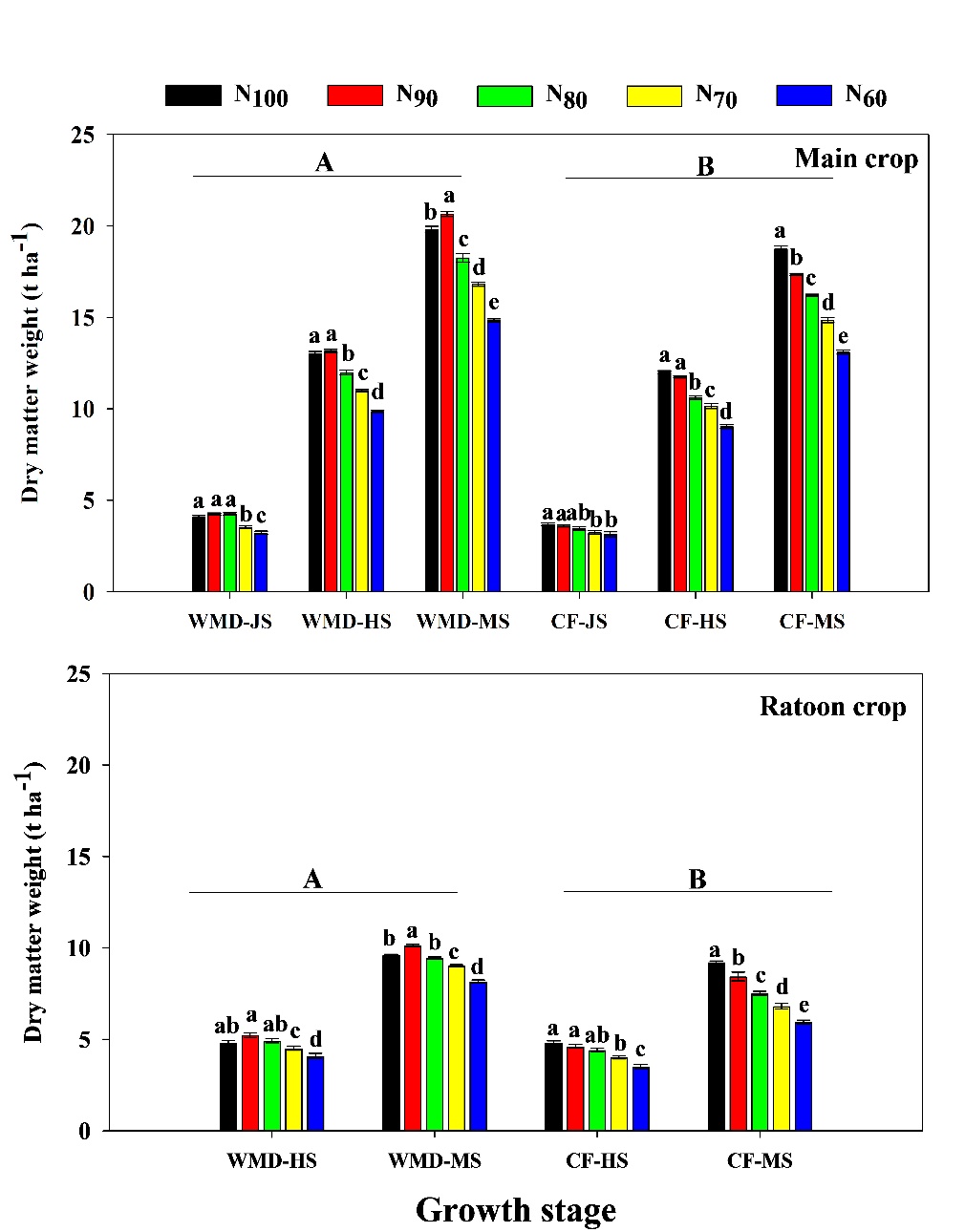 |
| --- |
| **Figure S4.** Effects of irrigation regime and N application rate on the DWA of main crop (A) and ratoon crop (B) in 2022. Note: CF, conventional flooding; WMD, alternate wetting and moderate soil drying irrigation. N_100_, N_90_, N_80_, N_70_ and N_60_ represent 100%, 90%, 80%, 70% and 60% N application rates, respectively. JS, HS and MS represent jointing stage, full heading stage and maturity stage. Different lowercase letters indicate significant differences among treatments at same growth stage, and different capital letters indicate significant differences between irrigation regime (at the 5% probability level according to the LSD test). |

## Supplementary Tables

**Table S1.** The interannual variance analysis in grain yield, soil nutrient content, and CEC across two seasons.

| Source of variation | df | Yield of main crop | Yield of ratoon crop | N | P | K | CEC |
| --- | --- | --- | --- | --- | --- | --- | --- |
| Year (Yr) | 1 | 2.67 | 3.55 | 2.39 | 15.42 | 1.96 | 0.15 |
| Irrigation regime (I) | 1 | 353.15** | 164.83** | 15306.27** | 68.04** | 31.93** | 794.08** |
| Nitrogen treatment (N) | 4 | 206.12** | 94.94** | 114.1** | 207.66** | 301.09** | 62.55** |
| Yr × I | 1 | 0.21 | 0.18 | 4.96 | 1.09 | 7.02 | 0.4 |
| Yr × N | 4 | 0.23 | 0.33 | 2.83* | 6.12** | 10.9** | 2.54 |
| N×I | 4 | 7.05** | 2.45 | 4.09** | 4.28** | 5.15** | 3.09* |
| Yr × I× N | 4 | 0.66 | 0.8 | 3.36* | 1.99 | 14.04** | 4.3** |

Note: N, P, K and CEC represent hydrolyzable N, available phosphorus, available potassium and cation exchange capacity, respectively. ** represents the significant difference at the 1% level according to LSD test, * represents the significant difference at the 5% level according to LSD test.

**Table S2.** The Interannual Variance Analysis in DMW and LAI during key growth stages of main and ratoon seasons.

| Source of variation | df | DMW | | | | LAI | | | |
| --- | --- | --- | --- | --- | --- | --- | --- | --- | --- |
|  |  | Full heading of MS | Maturity of MS | Full heading of RS | Maturity of RS | Full heading of MS | Maturity of MS | Full heading of RS | Maturity of RS |
| Year (Yr) | 1 | 12.15 | 0.92 | 8.1 | 7.67 | 10.55 | 8.22 | 14.53 | 1.56 |
| Irrigation regime (I) | 1 | 785.62** | 2006.08** | 305.21** | 577.44** | 282.89** | 286.08** | 5444.97** | 408.03** |
| Nitrogen treatment (N) | 4 | 383.41** | 1145.71** | 259.05** | 170.75** | 713.07** | 274.51** | 131.66** | 171.88** |
| Yr × I | 1 | 3.32 | 1.24 | 2.52 | 2.26 | 0.89 | 2.58 | 57.8** | 0.35 |
| Yr × N | 4 | 0.6 | 2.03 | 4.5** | 3.44* | 0.13 | 0.68 | 1.48 | 0.96 |
| N×I | 4 | 5.36** | 30.78** | 13.91** | 12.92** | 3.83* | 22.59** | 3.67* | 7.25** |
| Yr × I× N | 4 | 0.9 | 0.14 | 1.23 | 3.82* | 1.03 | 0.29 | 1.62 | 6.02** |

Note: DMW, dry matter weight; LAI, leaf area index. MS, main season; RS, ratoon season. ** represents the significant difference at the 1% level according to LSD test, * represents the significant difference at the 5% level according to LSD test.

**Table S3.** The interannual variance analysis in ROA during maturity stages of main and ratoon seasons.

| Source of variation | df | ROA | |
| --- | --- | --- | --- |
|  |  | Maturity of MS | Maturity of RS |
| Year (Yr) | 1 | 0.02 | 0.07 |
| Irrigation regime (I) | 1 | 413.4** | 186.6** |
| Nitrogen treatment (N) | 4 | 197.11** | 112.45** |
| Yr × I | 1 | 1.25 | 1.96 |
| Yr × N | 4 | 0.61 | 0.84 |
| N×I | 4 | 10.03** | 4.93** |
| Yr × I× N | 4 | 0.42 | 0.38 |

Note: ROA, root oxidation activity. MS, main season; RS, ratoon season. ** represents the significant difference at the 1% level according to LSD test.

**Table S4.** Effects of irrigation regime and N application rate on the grain yield of main crop in 2022.

| Treatment | | Panicles (10^4^ ha^-1^) | Spike Panicle^-1^ | Total spikelets (10^6^ ha^-1^) | Filled grain rate (%) | 1000-grain weight (g) | Grain yield (t ha^-1^) |
| --- | --- | --- | --- | --- | --- | --- | --- |
| WND | N_100_ | 311.45±3.05a | 157.33±2.77a | 490.00±3.83a | 72.45±0.85c | 28.43±0.07a | 10.10±0.22b |
|  | N_90_ | 309.88±4.68a | 156.25±1.05a | 484.19±6.45b | 76.13±1.43b | 28.77±0.47a | 10.60±0.60a |
|  | N_80_ | 296.77±2.53c | 152.60±1.40b | 452.87±8.02c | 76.51±0.31b | 28.78±0.58a | 10.00±0.07b |
|  | N_70_ | 266.73±6.77e | 147.15±1.35c | 392.49±4.94e | 77.06±0.26a | 28.83±0.43a | 8.72±0.02e |
|  | N_60_ | 256.26±7.36g | 140.02±2.68d | 358.82±3.45g | 78.92±0.08a | 28.82±0.52a | 8.22±0.22g |
|  | **Mean** | **288.22±1.16A** | **150.67±0.89A** | **435.68±1.54A** | **76.21±0.21A** | **28.73±0.39A** | **9.53±0.14A** |
| CF | N_100_ | 311.41±2.79a | 155.53±3.37a | 484.33±14.84b | 71.24±0.76c | 28.31±0.19a | 9.83±0.47c |
|  | N_90_ | 301.12±3.12b | 151.85±0.85b | 457.25±7.29c | 72.25±0.25c | 28.33±0.43a | 9.36±0.32d |
|  | N_80_ | 287.11±1.91d | 146.02±2.22c | 419.25±9.17d | 72.23±0.57c | 28.41±0.41a | 8.62±0.24f |
|  | N_70_ | 260.01±8.89f | 144.33±0.63cd | 375.27±11.20f | 72.63±0.37c | 28.41±0.01a | 7.66±0.27h |
|  | N_60_ | 243.09±2.39h | 138.52±1.62e | 336.72±7.24h | 74.52±0.72b | 28.41±0.31a | 7.13±0.30i |
|  | **Mean** | **280.55±0.85B** | **147.25±0.39B** | **414.56±0.47B** | **72.57±0.15B** | **28.37±0.19A** | **8.52±0.03B** |
| I | | 50.76* | 17.43 | 1196.84** | 820.95** | 10.70 | 800.3** |
| N | | 162.67** | 66.86** | 240.29** | 51.4** | 0.94 | 110.33** |
| I×N | | 1.34 | 1.54 | 1.81 | 7.84** | 0.31 | 4.56* |

Note: CF, conventional flooding; WMD, alternate wetting and moderate soil drying irrigation. N_100_, N_90_, N_80_, N_70_ and N_60_ represent 100%, 90%, 80%, 70% and 60% N application rates, respectively. I, irrigation regime; N, N application rate. Data in a column followed by different lower-case letters indicate significant differences at the 5% probability level according to the LSD test. Means followed by different upper-case letters indicate significant differences between the three nitrogen fertilizer levels at the 5% probability level according to the LSD test. ** represents the significant difference at the 1% level according to LSD test, * represents the significant difference at the 5% level according to LSD test.

Table S5. Effects of irrigation regimes and N application rate on the grain yield of ratoon crop in 2022.

| Treatment | | Panicles (10^4^ ha^-1^) | Spike Panicle^-1^ | Total spikelets (10^6^ ha^-1^) | Filled grain rate (%) | 1000-grain weight (g) | Grain yield (t ha^-1^) |
| --- | --- | --- | --- | --- | --- | --- | --- |
| WND | N_100_ | 438.16±7.09a | 67.11±1.94a | 294.05±13.26a | 65.43±e | 27.72±0.12a | 5.33±0.17a |
|  | N_90_ | 430.62±2.00b | 66.81±0.46a | 287.70±3.32b | 67.23±d | 28.07±0.67a | 5.43±0.03a |
|  | N_80_ | 415.03±3.10c | 64.03±1.29b | 265.72±3.39d | 67.35±d | 28.12±0.62a | 5.03±0.09b |
|  | N_70_ | 380.12±7.96d | 59.52±1.10c | 226.25±8.92f | 68.14±d | 28.12±0.82a | 4.34±0.23f |
|  | N_60_ | 327.86±16.94f | 56.82±6.71d | 186.30±31.64g | 75.42±a | 28.82±0.72a | 4.05±1.11e |
|  | **Mean** | **398.35±4.58A** | **62.86±1.01A** | **252.00±5.45A** | **68.71±B** | **28.17±0.59A** | **4.85±0.26A** |
| CF | N_100_ | 417.62±12.58c | 64.36±1.66b | 276.33±15.64c | 65.71±e | 28.05±0.25a | 4.95±0.16b |
|  | N_90_ | 385.22±11.00d | 63.06±2.27b | 248.30±15.98e | 67.00±d | 28.15±0.05a | 4.58±0.15d |
|  | N_80_ | 356.23±7.98e | 61.10±1.08c | 217.89±8.71f | 68.75±d | 28.57±0.57a | 4.27±0.25f |
|  | N_70_ | 325.11±10.79f | 57.55±0.30d | 186.73±7.19g | 71.15±c | 28.65±0.15a | 3.81±0.11g |
|  | N_60_ | 309.33±11.03g | 54.23±3.50e | 161.86±17.19h | 73.66±b | 28.75±0.37a | 3.55±0.45h |
|  | **Mean** | **358.70±1.24B** | **60.06±0.19B** | **218.22±0.79B** | **69.26±A** | **28.43±0.17A** | **4.25±0.22B** |
| I | | 270.69** | 5329.98** | 393.77** | 3.24 | 475.0** | 13.45 |
| N | | 120.60** | 19.70** | 62.11** | 13.14** | 4.96** | 43.92** |
| I×N | | 4.10* | 0.22 | 1.06 | 2.13 | 0.75 | 2.88 |

Note: CF, conventional flooding; WMD, alternate wetting and moderate soil drying irrigation. N_100_, N_90_, N_80_, N_70_ and N_60_ represent 100%, 90%, 80%, 70% and 60% N application rates, respectively. I, irrigation regime; N, N application rate. Data in a column followed by different lower-case letters indicate significant differences at the 5% probability level according to the LSD test. Means followed by different upper-case letters indicate significant differences between the three nitrogen fertilizer levels at the 5% probability level according to the LSD test. ** represents the significant difference at the 1% level according to LSD test, * represents the significant difference at the 5% level according to LSD test.

**Table S6.** Effects of irrigation regime and N application rate on the soil chemical properties in 2022.

| Treatment | | N（mg/kg） | P (mg/kg) | K (mg/kg) | CEC (mol/kg) |
| --- | --- | --- | --- | --- | --- |
|  |  |  |  |  |  |
| WND | N_100_ | 100.25±3.28a | 27.33±1.48a | 158.65±0.67a | 16.85±0.05a |
|  | N_90_ | 96.26±1.95a | 23.56±1.07b | 154.52±0.20b | 16.20±0.10b |
|  | N_80_ | 95.03±3.30ab | 23.02±0.67b | 146.23±2.03c | 15.80±0.12c |
|  | N_70_ | 89.26±3.29cd | 20.22±1.50c | 144.51±1.14cd | 15.30±0.06d |
|  | N_60_ | 84.54±4.15de | 18.63±0.27d | 142.36±0.87de | 14.80±0.28e |
|  | **Mean** | **93.07±1.53A** | **22.55±0.57A** | **149.25±0.45A** | **15.79±0.07A** |
| CF | N_100_ | 90.26±1.07bc | 24.50±0.84b | 155.63±0.62b | 14.61±0.20e |
|  | N_90_ | 81.85±1.53e | 20.54±2.56c | 154.33±1.00b | 14.20±0.05f |
|  | N_80_ | 74.52±1.74f | 19.56±1.01cd | 142.22±0.20de | 14.00±0.21fg |
|  | N_70_ | 72.54±2.98fg | 17.33±0.07e | 140.36±1.94ef | 13.81±0.15g |
|  | N_60_ | 67.95±3.94g | 16.51±1.66f | 138.75±2.19f | 13.82±0.35g |
|  | **Mean** | **77.42±0.06B** | **19.69±0.20B** | **146.26±0.54B** | **14.08±0.01B** |
| I | | 454.57** | 178.37** | 8.69 | 725.84** |
| N | | 53.88** | 32.91** | 206.57** | 55.87** |
| I×N | | 3.68* | 0.19 | 2.45 | 10.53** |

Note: CF, conventional flooding; WMD, alternate wetting and moderate soil drying irrigation. N, P, K and CEC represent hydrolyzable N, available phosphorus, available potassium and cation exchange capacity, respectively. N_100_, N_90_, N_80_, N_70_ and N_60_ represent 100%, 90%, 80%, 70% and 60% N application rates, respectively. I, irrigation regime; N, N application rate. Data in a column followed by different lower-case letters indicate significant differences at the 5% probability level according to the LSD test. Means followed by different upper-case letters indicate significant differences between the three nitrogen fertilizer levels at the 5% probability level according to the LSD test. ** represents the significant difference at the 1% level according to LSD test, * represents the significant difference at the 5% level according to LSD test.

**Table S7.** Effects of irrigation regime and N application rate on the N utilization rate of ratoon rice in 2022.

| Treatment | | NPFP (g/g) | NAE (g/g) |
| --- | --- | --- | --- |
|  |  |  |  |
| WND | N_100_ | 32.73±0.19g | 14.89±0.01b |
|  | N_90_ | 37.61±0.34f | 17.83±0.07a |
|  | N_80_ | 39.09±0.22e | 17.54±0.14a |
|  | N_70_ | 49.48±1.23b | 17.66±1.85a |
|  | N_60_ | 53.88±0.95a | 17.02±0.49a |
|  | **Mean** | **42.56±0.31A** | **16.99±0.23A** |
| CF | N_100_ | 30.85±1.19h | 13.66±0.17bc |
|  | N_90_ | 32.63±0.39g | 13.15±0.60cd |
|  | N_80_ | 33.78±1.18g | 12.07±0.27de |
|  | N_70_ | 43.04±0.50d | 11.71±0.34ef |
|  | N_60_ | 46.43±1.78c | 10.44±1.89f |
|  | **Mean** | **37.35±0.54B** | **12.21±0.65B** |
| I | | 288.67** | 375.25** |
| N | | 389.08** | 3.79* |
| I×N | | 7.11** | 9.80** |

Note: CF, conventional flooding; WMD, alternate wetting and moderate soil drying irrigation. NPFP, N partial factor productivity; NAE, N agronomic efficiency. N_100_, N_90_, N_80_, N_70_ and N_60_ represent 100%, 90%, 80%, 70% and 60% N application rates, respectively. I, irrigation regime; N, N application rate. Data in a column followed by different lower-case letters indicate significant differences at the 5% probability level according to the LSD test. Means followed by different upper-case letters indicate significant differences between the three nitrogen fertilizer levels at the 5% probability level according to the LSD test. ** represents the significant difference at the 1% level according to LSD test, * represents the significant difference at the 5% level according to LSD test.
